# Cross-cultural adaptation and validation of the Person-centered Practice Inventory-Staff for Brazilian culture*

**DOI:** 10.1590/1518-8345.7803.4665

**Published:** 2025-11-17

**Authors:** Juliana Andrioli Nucci, Edinêis de Brito Guirardello, Ariane Polidoro Dini

**Affiliations:** 1 Universidade Estadual de Campinas, Faculdade de Enfermagem, Campinas, SP, Brazil

**Keywords:** Translating Process, Transcultural Studies, Validation Studies, Patient-Centered Care, Psychometrics, Reproducibility of Results

## Abstract

to translate, cross-culturally adapt and validate the Person-centered Practice Inventory-Staff into Brazilian Portuguese.

the methodological study followed the stages of translation, synthesis, back-translation, expert evaluation and pre-testing. Content validity was assessed using the Content Validity Index and the Content Validity Ratio. Convergent and discriminant construct validity was verified using confirmatory factor analysis. Internal consistency was checked by Cronbach’s alpha, Composite Reliability and McDonald’s Omega.

15 professionals took part in the content validation and 307 in the evaluation of the construct and reliability. Content validity obtained values above 0.87 for the Content Validity Index and 0.73 for the Content Validity Ratio. The confirmatory factor analysis distributed the 59 items in the 17 original domains, with average variance extracted greater than 0.49 and factor loadings greater than 0.61. Internal consistency showed Cronbach’s alpha between 0.56 and 0.85; composite reliability between 0.75 and 0.89; and McDonald’s Omega between 0.61 and 0.88.

the Brazilian version of the Person-centered Practice Inventory-Staff demonstrated satisfactory validity and reliability to assess the perception of person-centered care practice among nursing professionals in the hospital context.

## Highlights

**(1)** The PCPI-S-BV instrument raises awareness of person-centered care. **(2)** The PCPI-S-BV, validated in European countries, will allow for international comparability. **(3)** The Brazilian version of the PCPI-S-BV shows evidence of validity and reliability. **(4)** The PCPI-S-BV instrument contributes to the delivery of safer care.

## Introduction

In the quest for excellence in safety standards and quality of care, health managers and politicians have been looking for new strategies to sensitize the professionals responsible for care, encouraging the adoption of a unique and humanized approach^(1)^.

Person-centered care (PCC) is built on valuing the perception and expectations not only of the person receiving care, but of everyone involved, i.e. family members, caregivers and health professionals^(2)^. PCC is a care practice established by the formation and promotion of healthy relationships between caregivers, service users and people who are significant in their lives. It is underpinned by values of respect for people, individual right to self-determination, mutual respect and understanding, and is enabled by cultures of empowerment that promote continuous approaches to the development of practice^(3)^.

Despite the relevance of PCC, its adherence faces challenges at a global level, mainly because it is confronted with the conventional biomedical model, cultural factors linked to organizations, the care environment, the profile of professionals, as well as the lack of reliable instruments to help monitor and evaluate practice in services^(2,4-5)^. From this perspective, the attention of the scientific community has focused on exploring the theoretical basis of PCC, in order to define concepts and definitions in a consensual and precise way^(2)^.

McCormack and McCance’s theoretical framework presents a theoretical structure for PCC, known as the Person-centered Practice Framework (PCPF). This is described as a mid-range theory supported by four constructs: (1) prerequisites, which address the characteristics of the professional; (2) the care environment, which refers to the context in which care is provided; (3) care processes, which focus on how care is provided; and (4) person-centered outcomes, which focus on achieving effective person-centered outcomes. According to the theory, professional attributes are considered essential for managing a care environment capable of effectively delivering person-centered outcomes^(6)^.

The Person-centered Practice Inventory-Staff (PCPI-S) was developed with the aim of investigating how health service teams and users perceive the perspectives of PCC. From the perspective of the professionals, this instrument aims to provide organizations and managers with support in drawing up a care plan that has the person as its central focus^(7)^.

The PCPI-S aims to analyze professionals’ perceptions of person-centered practice^(7)^. This instrument comprises 59 items distributed over 17 domains that enable the multi-professional team to self-assess their person-centered practice and has been validated for application in various care contexts. It is an instrument that has shown content validity, construct validity and internal consistency in Norway, Austria, Malaysia, Korea, Germany and Portugal^(7-14)^.

In Brazil, the evaluation of PCC is in its infancy and there is a lack of instruments to assess professionals’ perceptions of this practice. The aim of this study was to translate, cross-culturally adapt and validate the Person-centered Practice Inventory-Staff into Brazilian Portuguese.

## Method

### Study design

This methodological study followed the following stages: translation, synthesis of translations, back-translation, analysis by a committee of experts, testing of the final version and submission to the author of the original instrument. Sequentially, the construct validity and internal consistency of the PCPI-S were analyzed^(15-18)^.

### Period

The data collection period took place after the study was approved by the Research Ethics Committee between October 2022 and December 2023.

### Translation and cross-cultural adaptation

The first stage consisted of translating the original version of the instrument from English into Brazilian Portuguese by two independent translators with experience and fluency in both languages, with Portuguese as their mother tongue. Only one of the translators was informed about the concepts and objectives of the PCPI-S, so that not knowing this information could allow the second translator to be more sensitive to detecting different meanings of the original instrument^(15)^.

The two translated versions were called T1 and T2 and were compared with the original instrument and synthesized, creating a single version, called the synthesis version of the translations (T12)^(15)^. This stage was carried out by a third independent translator, whose mother tongue was Portuguese and who was fluent in English^(15)^.

The back-translation stage involved translating the synthesis version (T12) back into the original language, generating two versions (RT1 and RT2). This stage was carried out by two independent translators whose mother tongue corresponded to that of the original instrument (English). At this stage, the professionals were not informed about the objectives of the PCPI-S^(15)^.

Once the translation, synthesis and back-translation stages had been completed, all the versions (T1, T2, T12, RT1 and RT2) were evaluated by the expert committee to develop the pre-final version of the PCPI-S^(15)^. For the composition of the expert committee, members were invited based on a curriculum analysis on the Lattes Platform, and professionals with experience in the translation and adaptation of instruments and involvement in research related to person-centered care, safety management and quality of care were intentionally selected. The invitation was sent by e-mail. After acceptance, a link was provided to access the instrument and the equivalence analysis form.

The process of analyzing the translated and back-translated versions of the PCPI-S by the expert committee made it possible to compare the initial translations (T1 and T2), the synthesis version (T12) and the back-translations (RT1 and RT2) and to examine the semantic, idiomatic, cultural and conceptual equivalences, as well as its content validity^(15)^.

The content validity of the PCPI-S was analyzed using the Content Validity Index (CVI) and the Content Validity Ratio (CVR)^(16-17)^.

To calculate the CVI, a four-point ordinal scale was used, with the following options: (1) Not relevant or unclear; (2) Needs major revision to be relevant or clear; (3) Needs minor revision to be relevant or clear; or (4) Relevant and representative. The CVI is calculated by adding the number of “3” and “4” answers divided by the total number of respondents^(16)^.

To assess the relevance of the PCPI-S items, the Content Validity Ratio (CVR) was used, in which experts assessed the instrument’s items using a three-point ordinal scale: 1) Unnecessary item; 2) Useful but unnecessary item; 3) Essential item^(16)^.

For content validity, considering the number of participants in the evaluation, the following values were considered acceptable: CVI greater than 0.80 and CVR greater than 0.54^(16)^.

After analyzing all the translations of the PCPI-S items and the committee’s suggestions, the pre-final version of the PCPI-S was obtained for the pre-test procedure^(15)^. The pre-test stage comprised the last phase of the translation and cross-cultural adaptation process and consisted of applying the pre-final version of the instrument to a sample of 30 subjects, care professionals, including nursing technicians and nurses. The professionals were selected intentionally by indication of the area supervisors of the sectors corresponding to the field of study. The invitation to take part in the study was sent by email and instant messaging application, provided by the supervisors. After agreeing to take part in the research, the professionals were given access to the link to the Informed Consent Form, which was filled in before accessing the form referring to the content of the pre-test stage. The professionals were instructed to evaluate the statements in the instrument in terms of how easy they were to understand and the clarity of the content, registering, where necessary, suggestions for improving the content.

After the pre-test stage, an e-mail was sent to the authors of the PCPI-S containing all the versions of the cross-cultural adaptation process, as well as the Brazilian version of the instrument.

### Analysis of the measurement properties of the PCPI-S Brazilian version

The PCPI-S was developed based on the theoretical structure of the PCP-F, which addresses the therapeutic relationship between professionals, the person being cared for, their family and caregivers^(6-7)^.

The instrument has 59 items, distributed over 17 domains guided by three constructs^(7)^. The domains related to professional prerequisites include professional competence (items 1, 2 and 3), the development of interpersonal skills (items 4, 5, 6 and 7), professional commitment to the job (items 8, 9, 10, 11 and 12), self-knowledge (items 13, 14 and 15) and clarity of beliefs and values (items 14, 15, 16, 17 and 18)^(7)^. Aspects related to the care environment domain include the ability to combine skills (items 19, 20 and 21), the exercise of sharing decision-making (items 22, 23, 24 and 25), the establishment of effective interprofessional relationships (items 26, 27 and 28), the sharing of power (items 29, 30, 31 and 32), the potential for innovation and risk-taking (items 33, 34 and 35), characteristics of the physical environment (items 36, 37 and 38), organizational support systems (items 39, 40, 41, 42 and 43) and the management of beliefs and values (items 44, 45, 46 and 47)^(7)^. The domains that address person-centered processes involve shared decision-making (items 48, 49 and 50), strategies for engaging the person (items 51, 52 and 53), a supportive attitude (items 54, 55 and 56) and, finally, the provision of holistic care (items 57, 58 and 59)^(7)^.

The items are answered using a five-point ordinal scale, the options for which are: totally disagree (1), disagree (2), neutral (3), agree (4) and totally agree (5)^(7)^.

The score is obtained by calculating the average score for each domain, adding up the score for all the items in the domain and dividing the total by the number of items in the domain^(7)^. A high average percentage of responses indicates a positive perception of practice in the care environment^(7)^.

The analysis of the construct validity and reliability of the Brazilian version of the PCPI-S was carried out on a sample of 307 nursing professionals from a large university hospital.

The site of the data collection was a general teaching hospital in the interior of the state of São Paulo, with a physical capacity of 403 beds distributed among different specialties and which provides specialized high-complexity care funded by the Unified Health System (SUS).

To collect and manage the data, we used the Google Forms^®^ form and an Excel^®^ spreadsheet to store the database.

All nurses and nursing technicians employed at the institution for at least six months were considered eligible for the study. Exclusion criteria were related to leave of absence, vacation or sick leave during the data collection period.

The invitation to take part in the study was sent by email and instant messaging application, provided by the supervisors. After agreeing to take part in the study, the professionals were given access to the link to the Free and Informed Consent form, which they filled in before accessing the Google Forms^®^ form.

### Data processing and analysis

A descriptive analysis was carried out on the sociodemographic and professional characteristics of the subjects taking part in the study. The percentages of the variables were calculated from the data collected on the Google Forms^®^ platform.

Construct validity was assessed using second-order confirmatory factor analysis. Structural equation models were used using Partial Least Squares (PLS) as the estimation method. Smart PLS 3.3.5 software was used to implement these analyses.

To assess construct validity, the model’s convergent validity was first assessed using the Average Variance Extracted (AVE) for each of the instrument’s 17 domains. This measure assesses the proportion of the variance of the items that is explained by the factor/domain to which they belong. For convergent validity to be adequate, AVE values must be greater than 0.5^(17-18)^.

Discriminant validity was assessed using the Fornell-Larcker criterion^(17-18)^, which compares whether the square root of the AVE for a given domain is higher than the correlation values between the other domains^(17-18)^. Another criterion considered to assess discriminant validity was cross-load analysis. In this case, it was checked whether the factor loading of an item was higher in the domain to which it was originally allocated^(17-18)^, i.e. whether each of the 59 PCPI-S items had a factor loading greater than 0.5 within its respective 0.5 domain^(17-18)^.

Reliability was assessed based on internal consistency. For this, Cronbach’s alpha, composite reliability and McDonald’s Omega were considered, with values equal to or greater than 0.6 indicating satisfactory consistency^(17-18)^.

### Ethical aspects

The study was approved by the State University of Campinas (UNICAMP) Research Ethics Committee [Certificate of Submission for Ethical Appraisal (CAAE): 62881722.6.0000.5404] and the consent of all subjects was obtained by signing the Informed Consent Form, in accordance with National Health Card/Ministry of Health Resolution (CNS/MS) 466/12.

## Results

The process of translating and adapting the instrument went smoothly, complying with international recommendations. For the composition of the committee of experts, an invitation was sent to around twenty-two professionals by e-mail, and the completed form was returned by fifteen professionals.

The committee was made up exclusively of women, one of whom was a teaching nurse with experience in the translation and adaptation of instruments and experience in the field of care in health services belonging to the culture of origin of the instrument; nine teaching nurses with experience in the management of person-centered care, safety and quality of care, a doctoral nurse involved in studies related to patient safety, two linguists with experience in the process of translating and adapting instruments, a doctor specializing in quality management and patient safety and a teaching nurse who is a researcher in person-centered care. A round of analysis was carried out by the expert committee before the pre-test stage. At this stage, the CVI and CVR values were calculated for all the items in the instrument.

In the assessment of content validity, items 3, 5, 7, 8, 9, 11, 12, 18, 23, 28, 30, 31, 33, 35, 36, 38, 39, 40, 43, 44, 45, 46, 47, 48, 49, 50, 51, 55, 58 and 59 had a CVI of 1.0. Items 1, 2, 4, 6, 10, 13, 15, 16, 17, 19, 21, 22, 24, 25, 26, 27, 29, 41, 42, 53, 54 and 57 had a CVI of 0.93. The following items had a CVI of 0.87: 14, 20, 32, 34, 37, 52, 56.

With regard to CVR, items 1, 3, 8, 9, 10, 11, 12, 18, 23, 25, 28, 31, 37, 40, 41, 42, 43, 46, 47, 49, 50, 52, 54, 55, 58 had a value of 1.0. The following items had a CVR of 0.87: 2, 4, 5, 6, 7, 15, 16, 17, 19, 21, 22, 24, 26, 27, 29, 30, 32, 35, 36, 38, 39, 52, 53, 56, 57. Items 13, 14, 20, 33, 34 and 53 had a CVR of 0.73.

The experts made minor suggestions for changes to the items. Although all the items in the instrument reached a consensus on equivalence, some suggestions were considered to improve clarity. These suggestions were analyzed and accepted by the researchers. After incorporating the experts’ suggestions and making the necessary changes to the items, it was not necessary to carry out further rounds of evaluation, as the changes were only grammatical and did not interfere with the content of the instrument.

Thirty professionals took part in the pre-test, fifteen of them nurses and fifteen nursing technicians, who worked in the emergency unit and the clinical/surgical inpatient units. Most of the professionals were between 31 and 40 years old (85%), female (87%) and had more than ten years’ experience at the institution (84%). All the participants considered the instrument adequate for assessing the perception of centered care among professionals, classifying the items as understandable or partially understandable. The average time taken to complete the instrument was 15 minutes, ranging from 10 to 31 minutes.

At the end of the pre-test stage, there were no suggestions from the participants to change the items.

Of the 307 professionals who answered the questionnaire, 240 (78%) were women and 67 (22%) men. The average length of experience in the field was 10 years (85.1%). The predominant age group was between 41 and 50 (42.2%), while 69.8% declared themselves to be white. The most frequently cited level of education was *lato sensu* specialization (37.3%). The results of the convergent validity and reliability of the PCPI-S-BV are shown in Table 1.

**Table 1-:** Convergent validity of the factor model and internal consistency of the Brazilian version of the Person-centered Practice Inventory-Staff (n = 307). Campinas, SP, Brazil, 2023

**Domain**	AVE*	**Internal consistency**		
		**Composite Reliability**	**Cronbach’s alpha**	**McDonald’s Omega**
Professionally competent	0.53	0.77	0.56	0.61
Developed interpersonal skills	0.49	0.79	0.66	0.75
Commitment to the job	0.55	0.86	0.80	0.83
Knowing oneself	0.63	0.83	0.70	0.72
Clear beliefs and values	0.59	0.81	0.65	0.66
Combination of skills	0.60	0.82	0.67	0.68
Shared decision-making	0.62	0.87	0.80	0.84
Effective team relationships	0.72	0.89	0.81	0.83
Sharing power	0.61	0.86	0.79	0.81
Potential for innovation and risk-taking	0.50	0.75	0.50	0.51
The physical environment	0.59	0.81	0.66	0.68
Organizational support systems	0.63	0.89	0.85	0.88
Working with the patient’s beliefs and values	0.56	0.84	0.74	0.81
Shared decision-making	0.59	0.81	0.65	0.66
Engagement	0.62	0.83	0.70	0.70
Having a supportive presence	0.71	0.88	0.79	0.80
Providing holistic care	0.71	0.88	0.79	0.81

*Average Variance Extracted

The results of the discriminant validity of the PCPI-S-BV factor model, using the Fornell-Larcker Criterion, are shown in Table 2.

Table 3 shows the cross-loadings of the Person-centered Practice Inventory-Staff factor model.

**Table 2-:** Discriminant validity of the Person-centered Practice Inventory-Staff factor model, using the Fornell-Larcker Criterion* (n = 307). Campinas, SP, Brazil, 2023

**Domain**	**Domain**																
	**1.**	**2.**	**3.**	**4.**	**5.**	**6.**	**7.**	**8.**	**9.**	**10.**	**11.**	**12.**	**13.**	**14.**	**15.**	**16.**	**17.**
**1.**	**0.73**																
**2.**	0.49	**0.70**															
**3.**	0.41	0.59	**0.74**														
**4.**	0.28	0.41	0.42	**0.79**													
**5.**	0.41	0.49	0.52	0.44	**0.77**												
**6.**	0.30	0.49	0.48	0.36	0.51	**0.77**											
**7.**	0.39	0.30	0.44	0.34	0.49	0.40	**0.79**										
**8.**	0.32	0.29	0.34	0.29	0.43	0.21	0.49	**0.85**									
**9.**	0.38	0.33	0.40	0.30	0.50	0.32	0.57	0.69	**0.78**								
**10.**	0.53	0.43	0.50	0.39	0.49	0.45	0.56	0.49	0.64	**0.71**							
**11.**	0.59	0.49	0.49	0.41	0.46	0.47	0.41	0.39	0.46	0.54	**0.77**						
**12.**	0.32	0.34	0.36	0.32	0.42	0.32	0.56	0.66	0.71	0.56	0.49	**0.79**					
**13.**	0.41	0.47	0.60	0.39	0.50	0.45	0.39	0.41	0.45	0.57	0.60	0.47	**0.75**				
**14.**	0.45	0.39	0.47	0.32	0.44	0.37	0.39	0.33	0.38	0.49	0.54	0.39	0.71	**0.77**			
**15.**	0.38	0.53	0.54	0.41	0.45	0.55	0.35	0.33	0.40	0.50	0.57	0.37	0.67	0.65	**0.79**		
**16.**	0.30	0.49	0.55	0.34	0.41	0.48	0.28	0.35	0.35	0.45	0.51	0.37	0.64	0.61	0.65	**0.84**	
**17.**	0.37	0.51	0.56	0.39	0.42	0.48	0.25	0.28	0.27	0.47	0.51	0.30	0.66	0.56	0.63	0.73	**0.84**

Note: 1. professionally competent; 2. developed interpersonal skills; 3. committed to the job; 4. knowing oneself; 5. clear beliefs and values; 6. combination of skills; 7. shared decision-making; 8. effective team relationships; 9. power-sharing; 10. potential for innovation and risk-taking; 11. the physical environment; 12. supportive organizational systems; 13. working with the patient’s beliefs and values; 14. shared decision-making. Sharing power; 10. Potential for innovation and risk-taking; 11. The physical environment; 12. Supportive organizational systems; 13. Working with the patient’s beliefs and values; 14. Shared decision-making; 15. Engagement; 16. Having a supportive presence; 17. Providing holistic care

*Square root values of Average Variance Extracted highlighted

**Table 3-:** Discriminant Validity of the Person-centered Practice Inventory-Staff Factor Model, by means of Cross-Loadings (n = 307). Campinas, SP, Brazil, 2023

**Items**	**1**	**2**	**3**	**4**	**5**	**6**	**7**	**8**	**9**	**10**	**11**	**12**	**13**	**14**	**15**	**16**	**17**
**1**	**0.61**	0.32	0.27	0.17	0.27	0.33	0.21	0.23	0.24	0.27	0.21	0.16	0.27	0.17	0.21	0.17	0.17
**2**	**0.80**	0.41	0.49	0.26	0.25	0.36	0.24	0.20	0.27	0.38	0.29	0.20	0.28	0.23	0.29	0.28	0.33
**3**	**0.77**	0.42	0.51	0.25	0.37	0.30	0.37	0.24	0.29	0.40	0.37	0.23	0.35	0.24	0.34	0.24	0.30
**4**	0.45	**0.65**	0.37	0.22	0.36	0.32	0.13	0.16	0.18	0.22	0.25	0.13	0.24	0.17	0.34	0.30	0.32
**5**	0.39	**0.73**	0.42	0.19	0.32	0.34	0.17	0.11	0.21	0.25	0.28	0.18	0.27	0.23	0.33	0.28	0.34
**6**	0.35	**0.77**	0.47	0.35	0.35	0.37	0.29	0.29	0.29	0.41	0.45	0.30	0.42	0.38	0.44	0.48	0.42
**7**	0.32	**0.65**	0.39	0.38	0.37	0.34	0.21	0.22	0.23	0.29	0.35	0.30	0.36	0.28	0.34	0.28	0.33
**8**	0.45	0.44	**0.67**	0.31	0.29	0.28	0.20	0.18	0.22	0.27	0.28	0.13	0.28	0.20	0.36	0.35	0.33
**9**	0.33	0.41	**0.74**	0.28	0.38	0.27	0.34	0.26	0.30	0.38	0.41	0.32	0.54	0.47	0.42	0.48	0.50
**10**	0.46	0.42	**0.74**	0.32	0.41	0.31	0.35	0.23	0.23	0.41	0.40	0.26	0.46	0.37	0.40	0.39	0.40
**11**	0.47	0.49	**0.79**	0.34	0.44	0.45	0.35	0.30	0.37	0.39	0.32	0.32	0.49	0.34	0.41	0.43	0.44
**12**	0.52	0.44	**0.77**	0.32	0.40	0.46	0.39	0.26	0.34	0.38	0.42	0.28	0.45	0.35	0.41	0.38	0.38
**13**	0.24	0.31	0.35	**0.83**	0.34	0.23	0.34	0.27	0.25	0.32	0.33	0.28	0.34	0.27	0.34	0.26	0.30
**14**	0.21	0.33	0.26	**0.78**	0.32	0.23	0.19	0.22	0.25	0.26	0.33	0.20	0.25	0.26	0.32	0.27	0.25
**15**	0.29	0.34	0.39	**0.76**	0.39	0.39	0.28	0.20	0.22	0.35	0.32	0.28	0.33	0.22	0.32	0.29	0.37
**16**	0.30	0.36	0.43	0.47	**0.75**	0.37	0.38	0.31	0.38	0.36	0.35	0.37	0.43	0.35	0.32	0.36	0.31
**17**	0.25	0.31	0.27	0.25	**0.74**	0.42	0.30	0.26	0.32	0.37	0.35	0.26	0.31	0.29	0.30	0.25	0.30
**18**	0.37	0.45	0.47	0.30	**0.81**	0.39	0.43	0.41	0.45	0.41	0.36	0.33	0.41	0.38	0.41	0.32	0.34
**19**	0.35	0.33	0.34	0.23	0.28	**0.76**	0.23	0.05	0.15	0.26	0.33	0.16	0.32	0.23	0.39	0.30	0.33
**20**	0.35	0.37	0.40	0.29	0.54	**0.83**	0.39	0.25	0.34	0.43	0.41	0.34	0.42	0.33	0.43	0.39	0.39
**21**	0.34	0.44	0.37	0.32	0.33	**0.73**	0.30	0.16	0.23	0.33	0.34	0.23	0.31	0.29	0.46	0.41	0.39
**22**	0.36	0.32	0.40	0.27	0.47	0.45	**0.78**	0.36	0.40	0.43	0.33	0.32	0.33	0.35	0.37	0.24	0.22
**23**	0.26	0.27	0.37	0.35	0.37	0.31	**0.83**	0.38	0.41	0.43	0.35	0.46	0.30	0.29	0.24	0.23	0.21
**24**	0.27	0.20	0.35	0.26	0.38	0.27	**0.80**	0.45	0.50	0.45	0.33	0.55	0.32	0.30	0.25	0.21	0.19
**25**	0.32	0.13	0.27	0.20	0.31	0.23	**0.76**	0.37	0.48	0.47	0.28	0.44	0.29	0.30	0.23	0.19	0.17
**26**	0.33	0.31	0.35	0.28	0.42	0.27	0.45	**0.90**	0.62	0.48	0.33	0.58	0.38	0.29	0.32	0.34	0.28
**27**	0.27	0.27	0.29	0.24	0.36	0.18	0.43	**0.91**	0.63	0.44	0.36	0.60	0.36	0.27	0.29	0.31	0.24
**28**	0.14	0.13	0.20	0.23	0.31	0.06	0.36	**0.74**	0.51	0.31	0.31	0.50	0.32	0.30	0.22	0.25	0.17
**29**	0.25	0.23	0.30	0.16	0.38	0.18	0.35	0.60	**0.78**	0.43	0.34	0.57	0.39	0.29	0.31	0.27	0.19
**30**	0.37	0.27	0.40	0.28	0.46	0.31	0.44	0.41	**0.73**	0.52	0.39	0.42	0.40	0.39	0.44	0.35	0.28
**31**	0.23	0.18	0.22	0.19	0.33	0.23	0.42	0.55	**0.77**	0.47	0.30	0.58	0.23	0.18	0.21	0.18	0.13
**32**	0.29	0.24	0.31	0.29	0.38	0.27	0.54	0.60	**0.83**	0.55	0.39	0.67	0.35	0.29	0.27	0.27	0.22
**33**	0.33	0.25	0.31	0.23	0.36	0.18	0.55	0.57	**0.69**	0.72	0.34	0.62	0.36	0.28	0.30	0.20	0.20
**34**	0.36	0.36	0.33	0.31	0.34	0.44	0.38	0.21	**0.36**	0.72	0.40	0.37	0.43	0.37	0.35	0.38	0.39
**35**	0.34	0.30	0.42	0.30	0.34	0.33	0.26	0.24	**0.30**	0.68	0.41	0.19	0.42	0.41	0.43	0.39	0.42
**36**	0.34	0.34	0.36	0.36	0.23	0.34	0.21	0.23	**0.31**	0.36	0.70	0.24	0.41	0.43	0.44	0.35	0.36
**37**	0.27	0.36	0.37	0.29	0.38	0.38	0.25	0.32	**0.34**	0.40	0.81	0.4	0.47	0.42	0.45	0.41	0.40
**38**	0.33	0.42	0.41	0.31	0.43	0.37	0.46	0.34	**0.40**	0.48	0.80	0.47	0.49	0.40	0.44	0.42	0.42
**39**	0.17	0.19	0.21	0.31	0.28	0.22	0.41	0.50	**0.49**	0.34	0.35	0.71	0.29	0.23	0.23	0.20	0.11
**40**	0.14	0.30	0.23	0.20	0.30	0.18	0.37	0.45	**0.47**	0.33	0.36	0.74	0.29	0.28	0.22	0.23	0.19
**41**	0.25	0.28	0.29	0.30	0.34	0.30	0.46	0.55	**0.57**	0.48	0.4	0.82	0.38	0.34	0.31	0.32	0.25
**42**	0.26	0.3	0.34	0.23	0.37	0.29	0.43	0.54	**0.66**	0.51	0.44	0.83	0.45	0.36	0.36	0.38	0.32
**43**	0.25	0.25	0.31	0.25	0.37	0.27	0.54	0.56	**0.60**	0.51	0.38	0.84	0.44	0.33	0.31	0.29	0.28
**44**	0.35	0.37	0.39	0.29	0.25	0.40	0.24	0.30	**0.33**	0.41	0.38	0.33	0.66	0.45	0.47	0.40	0.42
**45**	0.28	0.34	0.46	0.29	0.33	0.32	0.21	0.26	**0.29**	0.38	0.40	0.29	0.81	0.59	0.56	0.54	0.60
**46**	0.32	0.35	0.51	0.27	0.49	0.31	0.38	0.35	**0.33**	0.49	0.46	0.41	0.76	0.56	0.46	0.54	0.48
**47**	0.30	0.36	0.45	0.32	0.42	0.34	0.33	0.33	**0.39**	0.43	0.55	0.38	0.76	0.52	0.51	0.44	0.48
**48**	0.20	0.31	0.36	0.19	0.36	0.27	0.27	0.27	**0.25**	0.40	0.38	0.26	0.59	0.80	0.53	0.46	0.51
**49**	0.22	0.30	0.42	0.24	0.40	0.30	0.42	0.29	**0.33**	0.39	0.42	0.40	0.55	0.79	0.44	0.45	0.39
**50**	0.26	0.28	0.29	0.31	0.25	0.29	0.19	0.19	**0.29**	0.34	0.44	0.23	0.48	0.71	0.52	0.50	0.40
**51**	0.35	0.46	0.46	0.37	0.31	0.49	0.28	0.24	**0.26**	0.39	0.50	0.25	0.52	0.47	0.79	0.48	0.55
**52**	0.27	0.38	0.35	0.32	0.39	0.33	0.26	0.28	**0.34**	0.42	0.37	0.30	0.49	0.48	0.76	0.45	0.43
**53**	0.30	0.41	0.46	0.29	0.37	0.47	0.28	0.26	**0.35**	0.39	0.48	0.33	0.57	0.58	0.82	0.60	0.52
**54**	0.23	0.42	0.44	0.25	0.34	0.43	0.24	0.34	**0.33**	0.34	0.45	0.34	0.55	0.59	0.64	0.88	0.60
**55**	0.26	0.39	0.47	0.33	0.36	0.39	0.27	0.28	**0.33**	0.40	0.45	0.33	0.61	0.52	0.52	0.86	0.64
**56**	0.33	0.42	0.47	0.29	0.33	0.39	0.18	0.27	**0.22**	0.41	0.39	0.26	0.46	0.42	0.48	0.79	0.60
**57**	0.33	0.37	0.38	0.32	0.29	0.42	0.20	0.16	**0.15**	0.35	0.36	0.14	0.47	0.36	0.44	0.53	0.75
**58**	0.29	0.43	0.48	0.29	0.36	0.38	0.22	0.24	**0.21**	0.41	0.44	0.28	0.58	0.55	0.56	0.64	0.87
**59**	0.34	0.47	0.53	0.37	0.38	0.42	0.21	0.28	**0.30**	0.43	0.48	0.31	0.59	0.49	0.58	0.66	0.90

## Discussion

The process of cross-cultural adaptation of the PCPI-S-Brazilian version followed the internationally recommended stages^(15-19)^, without major challenges, due to the committee’s commitment to evaluating the 59 items, as well as the objective structure and theoretical framework of the instrument^(6-7)^.

Based on the assumption that reading, interpreting and completing an instrument generates not only data, but also instigates the subjects to reflect on their practice and subsequently move towards change in order to improve or adjust their performance, attention was paid throughout the process of translating and adapting the instrument to replicate the original construct^(15)^.

In order to stimulate self-analysis and raise awareness among the subjects, placing themselves in the situation presented by the statement, all the statements were readjusted, starting with the pronoun “I” and replacing the terms “I take” and “I use” with the terms “I dedicate” and “I usually”, in items 13 and 14 respectively.

In the analysis of the experts’ opinion, the term “challenge”, present in items 7 and 17, generated ambiguity, being interpreted by some in a pejorative way, associating it with competition and interpersonal conflicts - contrary to the proposed model of care. Considering that the statement aims to investigate the subject’s position in relation to the team in the face of divergent practices, it was decided, after consensus between the researchers, the expert committee and the linguist, to replace the term with “confrontation”. The term “ward rounds”, present in item 25, was translated as “nursing rounds”, an expression often used in European countries to describe the bedside shift change carried out by nursing professionals when changing shifts. However, this terminology is not common in Brazilian culture, which could lead to different interpretations among the participants. In view of this, it was decided to change the term to “bedside shift handover”, in order to make the statement easier to understand.

The content validation of the PCPI-S, internationally recognized as the most important stage in the process of validating instruments^(16)^, relied on the extensive experience and training of the participants on the expert committee. The evaluations of 15 professionals working in management, teaching and research were analyzed. As a result, the PCPI-P version obtained a CVI of over 0.87 and a CVR of over 0.73 for all items, exceeding the values recommended for analysis by 15 judges^(16)^.

After finalizing the adjustments suggested by the committee of experts, the pre-final version of the PCPI-S was drawn up and then submitted to a pre-test. The average response time to the instrument was 15 minutes, so the participants considered it adequate and understandable.

After completing the pre-test, the instrument’s measurement properties were assessed, which corresponded to the assessment of the convergent and discriminant construct validity and reliability of the PCPI-S.

In the confirmatory factor analysis, the AVE and cross-load values were higher than 0.5, confirming the convergent construct validity and maintaining 17 domains of the original PCPI-S^(17-18)^. Although only domain 2 “Developed interpersonal skills” had an AVE of 0.49, which is too close to 0.5 to suggest convergent validity, all the items in this domain had factor loadings higher than 0.65, indicating evidence of convergent validity in this domain as well^(17-18)^.

The analysis also shows evidence of discriminant validity, based on the Fornell-Larcker Criterion, which shows that the correlation values within each domain were higher than the correlations between the different domains^(17-18)^. With regard to reliability, the analysis of the instrument’s internal consistency indicates that, although Cronbach’s alpha showed values between 0.5 and 0.8, this coefficient is an estimate of the reliability of measuring a single construct common to all the items in a domain, provided that it is unidimensional and has similar factor loadings. As the items within the same domain showed multifactorial behavior, alternative reliability measures were carried out, such as the McDonald Omega, which ranged from 0.61 to 0.88, and the composite reliability assessment, which does not assume unidimensionality of the items and is therefore a more impartial measure. The composite reliability showed values between 0.77 and 0.89, indicating evidence of internal consistency of the PCPI-S-BV and reinforcing the instrument’s accuracy^(15,17)^.

Unlike the Malaysian translation^(10)^, which faced challenges in translation and adaptation due to the concern to maintain the interdisciplinarity of the instrument, as well as having been carried out in a primary care context with a multi-professional sample, the process of cross-cultural adaptation to the Brazilian scenario did not present any significant difficulties. In the Malaysian version, the use of exploratory factor analysis resulted in the exclusion of 6 of the 17 domains. In the adaptation to Brazil, it was possible to maintain the original structure of the instrument, in line with the other translations^(7-9,11-14)^.

Conceptually, the theoretical framework of person-centered practice argues that the perception of all the people involved in the care process should be considered and valued, encouraging harmonious relationships and consequently strengthening the bond between professionals, family members and patients^(6)^. Thus, the use of the PCPI-S-BV can sensitize health professionals to non-technical dimensions of care, such as the patient’s experience^(20-23)^. From this perspective, it is believed that the use of the instrument validated in this study favors a virtuous cycle of care among professionals, integrating the affective, cognitive, behavioral, moral and empathic dimensions of person-centered care. The interaction between these dimensions enables professionals to share and understand the feelings of the sick person, improving communication and promoting more humanized care. In this way, the desire to help is driven by a genuine internal motivation on the part of health professionals^(23)^.

Therefore, the main contribution of this study is the availability of the Brazilian version of the Person-centered Practice Inventory-Staff to raise awareness among health professionals and promote person- and family-centered care. The instrument’s validation in other countries^(8-14)^ indicates its credibility, enabling data comparability and the identification of strategies for developing professionals with clinical competencies and behavioral skills focused on person-centered care.

The original PCPI-S was developed to be applied in different care environments and aimed at multi-professional health teams. Thus, the main limitation of this study is that data collection for analyzing the measurement properties of the Brazilian version of the PCPI-S was carried out only with nursing professionals in a single hospital institution. Therefore, it is recommended that the Brazilian version of the PCPI-S be applied in different care settings, involving other professionals from the multi-professional team

## Conclusion

The Brazilian version of the Person-centered Practice Inventory-Staff showed evidence of validity and internal consistency to measure the perception of the practice of person-centered care among nursing professionals in the hospital setting.

The importance of investigating and validating the perception of other professional categories regarding person-centered care in a broader sample scenario is reiterated. Considering that the instrument can be applied to different professional categories and in different care contexts, it is important to explore different populations and environments.

## Data Availability

All data generated or analysed during this study are included in this published article.
