# Supplementary material for: Cross-cultural adaptation and validation of the Person-centered Practice Inventory-Staff for Brazilian culture
Source: Rev Lat Am Enfermagem. 2025 Nov 17;33:e4665. doi: 10.1590/1518-8345.7803.4665 (PMC12628370; doi:10.1590/1518-8345.7803.4665)
Supplement: Translation

# Translation: Adaptação transcultural e validação do *Person-centred Practice Inventory-Staff* para a cultura brasileira*

**DOI:** 10.1590/1518-8345.7803.4666

**Authors:** Juliana Andrioli Nucci, Edinêis de Brito Guirardello, Ariane Polidoro Dini

**Affiliations:** 1 Universidade Estadual de Campinas, Faculdade de Enfermagem, Campinas, SP, Brasil

**Keywords:** Processo de Tradução, Estudos Transculturais, Estudos de Validação, Assistência Centrada na Pessoa, Psicometria, Reprodutibilidade dos Testes

## Abstract

realizar a tradução, adaptação transcultural e validação do *Person-centred Practice Inventory-Staff* para língua portuguesa do Brasil.

estudo metodológico seguindo as etapas de tradução, síntese, retrotradução, avaliação por especialistas e pré-teste. A validade de conteúdo foi avaliada pelo Índice de Validade de Conteúdo e pela Razão de Validade de Conteúdo. A validade de construto convergente e discriminante foi verificada por meio de análise fatorial confirmatória. A consistência interna foi verificada pelo alfa de Cronbach, Confiabilidade Composta e Ômega de McDonald.

participaram 15 profissionais na validação de conteúdo e 307 na avaliação do construto e confiabilidade. A validade de conteúdo obteve valores acima de 0,87 para o Índice de Validade de Conteúdo e 0,73 para Razão de Validade de Conteúdo. A análise fatorial confirmatória distribuiu os 59 itens nos 17 domínios originais, com variância média extraída superior a 0,49 e cargas fatoriais superiores a 0,61. A consistência interna, apresentou alfa de Cronbach entre 0,56 e 0,85; confiabilidade composta entre 0,75 e 0,89; e Ômega de McDonald entre 0,61 e 0,88.

a versão brasileira do *Person-centred Practice Inventory-Staff* demonstrou validade e confiabilidade satisfatórias para avaliar a percepção da prática do cuidado centrado na pessoa, entre profissionais de enfermagem, no contexto hospitalar.

## Destaques

**(1)** Instrumento PCPI-S-VB permite a sensibilização quanto ao cuidado centrado na pessoa. **(2)** O PCPI-S-VB validado nos países europeus, permitirá a comparabilidade internacional. **(3)** A versão brasileira do PCPI-S-VB apresenta evidências de validade e confiabilidade. **(4)** O instrumento PCPI-S-VB contribui para a entrega de um cuidado mais seguro.

## Introdução

Em busca de atribuir excelência aos padrões de segurança e qualidade assistenciais, gestores e políticos de saúde têm buscado novas estratégias para sensibilizar os profissionais responsáveis pelo cuidado, incentivando na adoção de uma abordagem singular e humanizada^(1)^.

O cuidado centrado na pessoa (CCP), é construído a partir da valorização da percepção e expectativas não só da pessoa que recebe o cuidado, mas de todos os envolvidos, ou seja, familiares, cuidadores e profissionais da saúde^(2)^. O CCP é uma prática de cuidado estabelecida pela formação e promoção de relacionamentos saudáveis entre cuidadores, usuários de serviços e pessoas significativas para suas vidas. É sustentado por valores de respeito pelas pessoas, direito individual à autodeterminação, respeito mútuo e compreensão, sendo possibilitado por culturas de empoderamento que promovem abordagens contínuas ao desenvolvimento da prática^(3)^.

Apesar da relevância do CCP, a sua adesão enfrenta desafios em nível global, principalmente por defrontar-se com o modelo biomédico convencional, fatores culturais ligados às organizações, o ambiente de cuidado, o perfil dos profissionais, além da carência de instrumentos confiáveis que auxiliem no monitoramento e avaliação da prática nos serviços^(2,4-5)^. Nessa perspectiva, a atenção da comunidade científica concentrou-se em explorar a fundamentação teórica do CCP, no intuito de definir de forma consensual e precisa de conceitos e definições^(2)^.

O referencial teórico de McCormack e McCance apresenta uma estrutura teórica para o CCP, conhecida como *Person-centred Practice Framework* (PCPF). Ela é descrita como uma teoria de médio alcance sustentada por quatro construtos: (1) pré-requisitos, que abordam as características do profissional; (2) o ambiente de cuidado, que se refere ao contexto em que o cuidado é prestado; (3) processos de cuidado, que se concentram na forma como o cuidado é prestado; e (4) resultados centrados na pessoa, que se focam no alcance de resultados eficazes centrados na pessoa. De acordo com a teoria, os atributos do profissional são considerados imprescindíveis para a gestão de um ambiente de cuidado capaz de fornecer com eficácia resultados centrados na pessoa^(6)^.

Com o objetivo de investigar como as equipes e usuários dos serviços de saúde percebem as perspectivas do CCP, foi desenvolvido o *Person-centred Practice Inventory-Staff* (PCPI-S). Esse instrumento propõe oferecer, sob a perspectiva dos profissionais, subsídios às organizações e gestores para a elaboração de um plano de cuidados que tenha a pessoa, como foco central^(7)^.

O PCPI-S, tem como finalidade analisar a percepção dos profissionais quanto à prática centrada na pessoa^(7)^. Esse instrumento abrange 59 itens distribuídos em 17 domínios que possibilitam a autoavaliação da equipe multiprofissional quanto à sua prática centrada na pessoa, e foi validado para ser aplicado em diversos contextos assistenciais. Trata-se de um instrumento que apresentou validade de conteúdo, de construto e consistência interna na Noruega, Áustria, Malásia, Coreia, Alemanha e Portugal^(7-14)^.

No Brasil, a avaliação do CCP é incipiente e carece de instrumentos que possibilitem a avaliação da percepção dos profissionais em relação a essa prática. O objetivo foi realizar a tradução, adaptação transcultural e validação do *Person-centred Practice Inventory-Staff* para língua portuguesa do Brasil.

## Método

### Delineamento do estudo

Estudo metodológico que seguiu as seguintes etapas de tradução, síntese das traduções, retrotradução, análise por um comitê de especialistas, teste da versão final e submissão ao autor do instrumento original. Sequencialmente, foram analisadas a validade de construto e a consistência interna do PCPI-S^(15-18)^.

### Período

O período de coleta de dados sucedeu a aprovação do estudo pelo Comitê de Ética em Pesquisa entre outubro de 2022 e dezembro de 2023.

### Tradução e adaptação transcultural

A primeira etapa consistiu na tradução da versão original do instrumento da língua inglesa para a língua portuguesa do Brasil por dois tradutores independentes, com experiência e fluência em ambos os idiomas, tendo como língua materna o português. Apenas um dos tradutores foi informado sobre os conceitos e objetivos do PCPI-S, de forma que o desconhecimento dessas informações pudesse permitir que o segundo tradutor estivesse mais sensível para detectar diferentes significados do instrumento original^(15)^.

As duas versões traduzidas foram denominadas T1 e T2 e foram equiparadas com o instrumento original e sintetizadas, concebendo uma versão única, nomeada de versão síntese das traduções (T12)^(15)^. Essa etapa foi desenvolvida por um terceiro tradutor independente, que possuía o português como língua materna e fluência em inglês^(15)^.

A etapa de retrotradução compreendeu a tradução da versão síntese (T12) de volta para o idioma original, gerando duas versões (RT1 e RT2). Essa etapa foi realizada por dois tradutores independentes, cuja língua materna era correspondente a do instrumento original (inglês). Nessa fase os profissionais não foram informados sobre os objetivos do PCPI-S^(15)^.

Findadas as etapas de tradução, síntese e retrotradução, procedeu-se à avaliação de todas as versões (T1, T2, T12, RT1 e RT2) pelo comitê de especialistas para desenvolver a versão pré-final do PCPI-S^(15)^. Para a composição do comitê de especialistas, os membros foram convidados a partir da análise curricular na Plataforma Lattes, sendo selecionados de forma intencional profissionais com experiência na tradução e adaptação de instrumentos e envolvimento em pesquisas relacionadas ao cuidado centrado na pessoa, gestão de segurança e qualidade assistencial. O convite foi encaminhado por meio de correio eletrônico. Após o aceite, foi disponibilizado o *link* para acesso ao instrumento e formulário de análise das equivalências.

O processo de análise das versões traduzidas e retrotraduzidas do PCPI-S pelo comitê de especialistas possibilitou a comparação das traduções iniciais (T1 e T2), versão síntese (T12) e retro traduções (RT1 e RT2) e permitiu examinar as equivalências semântica, idiomática, cultural e conceitual, além de sua validade de conteúdo^(15)^.

A validade de conteúdo do PCPI-S foi analisada por meio do Índice de Validade de Conteúdo (IVC) e da Razão de Validade de Conteúdo (RVC)^(16-17)^.

Para o cálculo do IVC, considerou-se uma escala ordinal com quatro pontos, com as opções: (1) Não relevante ou não apresenta clareza; (2) Precisa de grande revisão para ser relevante ou apresentar clareza; (3) Precisa de pequena revisão para ser relevante ou apresentar clareza; ou (4) Relevante e representativo. O cálculo do IVC é realizado pelo somatório do número de respostas “3” e “4” dividido pelo número total de respondentes^(16)^.

Para avaliação da relevância dos itens do PCPI-S, utilizou-se a Razão de Validade de Conteúdo (RVC), na qual os especialistas avaliaram os itens do instrumento a partir de uma escala ordinal com três pontos, sendo: 1) Item desnecessário; 2) Item útil, mas desnecessário; 3) Item essencial^(16)^.

Para a validade de conteúdo, considerando o número de participantes na avaliação, foram considerados valores aceitáveis: IVC superior a 0,80 e RVC superior a 0,54^(16)^.

Após a análise de todas as versões de traduções dos itens do PCPI-S e sugestões do comitê, foi obtida a versão pré-final do PCPI-S para a realização do procedimento do pré-teste^(15)^. A etapa do pré-teste compreendeu a última fase do processo da tradução e adaptação transcultural e consistiu na aplicação da versão pré-final do instrumento em uma amostra de 30 sujeitos, profissionais da área assistencial, entre eles técnicos de enfermagem e enfermeiros. Os profissionais foram selecionados intencionalmente por meio da indicação dos supervisores de área dos setores correspondentes ao campo de estudo. O convite para a participação na pesquisa foi enviado por meio de correio eletrônico e aplicativo instantâneo de mensagem, fornecidos pelos supervisores. Após o aceite em participar da pesquisa, os profissionais tiveram acesso ao *link* do Termo de Consentimento Livre e Esclarecido, que foi preenchido antes do acesso ao formulário referente ao conteúdo da etapa do pré-teste. Os profissionais foram orientados a avaliar as afirmativas do instrumento quanto à facilidade de entendimento e clareza do conteúdo, registrando, quando necessário, sugestões de melhoria para o aprimoramento do conteúdo.

Após a etapa do pré-teste foi encaminhado um *e-mail* aos autores do PCPI-S contendo todas as versões do processo de adaptação transcultural, bem como a versão brasileira do instrumento.

### Análise das propriedades de medida do PCPI-S versão brasileira

O PCPI-S foi desenvolvido a partir da estrutura teórica do PCP-F que aborda a relação terapêutica entre os profissionais, a pessoa a ser cuidada, seus familiares e cuidadores^(6-7)^.

O instrumento possui 59 itens, distribuídos em 17 domínios guiados por três construtos^(7)^. Os domínios relacionados aos pré-requisitos profissionais incluem a competência profissional (itens 1, 2 e 3), o desenvolvimento de habilidades interpessoais (itens 4, 5, 6 e 7), o comprometimento profissional com o trabalho (itens 8, 9, 10, 11 e 12), o autoconhecimento (itens 13, 14 e 15) e a clareza de crenças e valores (itens 14, 15, 16, 17 e 18)^(7)^. Os aspectos relacionados ao domínio ambiente de cuidado incluem a capacidade de combinar habilidades (itens 19, 20 e 21), o exercício de partilhar a tomada de decisões (itens 22, 23, 24 e 25), o estabelecimento de relações interprofissionais eficazes (itens 26, 27 e 28), o compartilhamento do poder (itens 29, 30, 31 e 32), o potencial para inovação e tomada de riscos (itens 33, 34 e 35), características do ambiente físico (itens 36, 37 e 38), os sistemas organizacionais de apoio (itens 39, 40, 41, 42 e 43) e o manejo de crenças e valores (itens 44, 45, 46 e 47)^(7)^. Os domínios que abordam os processos centrados na pessoa envolvem a tomada de decisão compartilhada (itens 48, 49 e 50), estratégias de engajamento da pessoa (itens 51, 52 e 53), postura solidária (itens 54, 55 e 56) e pôr fim à prestação de cuidados holísticos (itens 57, 58 e 59)^(7)^.

As respostas aos itens são realizadas a partir de uma escala ordinal com cinco pontos, cujas opções são: discordo totalmente (1), discordo (2), neutro (3), concordo (4) e concordo totalmente (5)^(7)^.

O escore é obtido pelo cálculo da pontuação média de cada domínio, somando a pontuação de todos os itens referentes ao domínio, dividindo o total pelo número de itens do domínio^(7)^. Uma média alta da porcentagem de respostas indica uma percepção positiva da prática no ambiente de cuidado^(7)^.

A análise da validade de construto e a confiabilidade da versão brasileira do PCPI-S foi obtida por meio de sua aplicação em uma amostra de 307 profissionais de enfermagem de um hospital universitário de grande porte.

O local da coleta de dados foi um hospital geral, de ensino, no interior do estado de São Paulo, com capacidade física para 403 leitos distribuídos entre diferentes especialidades e que conta com atendimentos especializados de alta complexidade, financiados pelo Sistema Único de Saúde (SUS).

Para a realização da coleta e gerenciamento dos dados, utilizou-se do formulário *Google Forms* ^®^ e da planilha eletrônica do programa *Excel* ^®^ para o armazenamento do banco de dados.

Todos os enfermeiros e técnicos de enfermagem com vínculo empregatício na instituição por um período mínimo de seis meses foram considerados elegíveis para o estudo. Os critérios de exclusão foram relacionados a situações de afastamentos, férias, licença-saúde no período da coleta de dados.

O convite para a participação na pesquisa foi enviado por meio de correio eletrônico e aplicativo instantâneo de mensagem, fornecidos pelos supervisores. Após o aceite em participar da pesquisa, os profissionais tiveram acesso ao link do termo de Consentimento Livre e Esclarecido, que foi preenchido antes do acesso ao formulário *Google Forms*®.

### Tratamento e análise dos dados

A análise descritiva foi realizada a partir do levantamento das características sociodemográficas e profissionais dos sujeitos participantes da pesquisa. As porcentagens das variáveis foram calculadas a partir dos dados coletados na plataforma *Google Forms* ^®^.

A validade de construto se deu por meio da análise fatorial confirmatória de segunda ordem. Foram utilizados modelos de equações estruturais considerando como método de estimação o *Partial Least Squares* (PLS), ou mínimos quadrados parciais. Para a implementação dessas análises foi utilizado o *software Smart* PLS 3.3.5.

Para avaliação da validade de construto, primeiramente foi avaliada a validade convergente do modelo, por meio da *Average Variance Extracted* (AVE) para cada um dos 17 domínios do instrumento. Essa medida avalia a proporção da variância dos itens que é explicada pelo fator/domínio a que pertencem. Para que a validade convergente fosse adequada, os valores de AVE devem ser superiores a 0,5^(17-18)^.

A validade discriminante foi avaliada, por meio do critério de Fornell-Larcker^(17-18)^, o qual compara se a raiz quadrada da AVE para determinado domínio é superior aos valores de correlação entre os demais domínios^(17-18)^. Outro critério considerado para avaliar a validade discriminante foi a análise das cargas cruzadas. Nesse caso, verificou-se se a carga fatorial de um item era mais elevada no domínio ao qual foi originalmente alocado^(17-18)^, ou seja, se cada um dos 59 itens do PCPI-S apresentava carga fatorial superior a 0,5 dentro do seu respectivo domínio 0,5^(17-18)^.

A confiabilidade foi avaliada com base na consistência interna. Para isso, foram considerados o alfa de Cronbach, a confiabilidade composta e o Ômega de McDonald, sendo que valores iguais ou superiores a 0,6 indicam consistência satisfatória^(17-18)^.

### Aspectos éticos

O estudo foi aprovado pelo Comitê de Ética em Pesquisa da Universidade Estadual de Campinas (UNICAMP) [Certificado de Apresentação de Apreciação Ética (CAAE): 62881722.6.0000.5404] e foi obtido o consentimento de todos os sujeitos por meio da assinatura do Termo de Consentimento Livre e Esclarecido, conforme a Resolução Cartão Nacional de Saúde/Ministério da Saúde (CNS/MS) 466/12.

## Resultados

O processo de tradução e adaptação do instrumento foi realizado cumprindo as recomendações internacionais. Para a composição do comitê de especialistas, foi encaminhado o convite para cerca de 22 profissionais, por meio de correio eletrônico, obtendo o retorno do formulário preenchido de quinze profissionais.

O comitê foi composto exclusivamente por mulheres, sendo uma enfermeira docente, com experiência na tradução e adaptação de instrumentos e vivência no campo assistencial em serviços de saúde pertencentes a cultura de origem do instrumento; nove enfermeiras, docentes, com experiência na gestão do cuidado centrado na pessoa, segurança e qualidade assistencial, uma enfermeira doutoranda envolvida em estudos relacionados à segurança do paciente, duas linguistas com experiência no processo de tradução e adaptação de instrumentos, uma médica especialista em gestão de qualidade e segurança do paciente e uma enfermeira, docente, pesquisadora do cuidado centrado na pessoa. Foi realizada uma rodada de análise pelo comitê de especialistas, antes da etapa do pré-teste. Nessa etapa, foram calculados os valores de IVC e RVC para todos os itens do instrumento.

Na avaliação da validade de conteúdo, os itens 3, 5, 7, 8, 9, 11, 12, 18, 23, 28, 30, 31, 33, 35, 36, 38, 39, 40, 43, 44, 45, 46, 47, 48, 49, 50, 51, 55, 58 e 59 apresentaram IVC de 1,0. Apresentaram IVC de 0,93 os itens: 1, 2, 4, 6, 10, 13, 15, 16, 17, 19, 21, 22, 24, 25, 26, 27, 29, 41, 42, 53, 54, 57. Apresentaram IVC de 0,87 os itens: 14, 20, 32, 34, 37, 52, 56.

No que se refere à RVC, apresentaram valor de 1,0 os itens 1, 3, 8, 9, 10, 11, 12, 18, 23, 25, 28, 31, 37, 40, 41, 42, 43, 46, 47, 49, 50, 52, 54, 55, 58. Apresentaram RVC de 0,87 os itens: 2, 4, 5, 6, 7, 15, 16, 17, 19, 21, 22, 24, 26, 27, 29, 30, 32, 35, 36, 38, 39, 52, 53, 56, 57. Já os itens 13, 14, 20, 33, 34 e 53 apresentaram RVC de 0,73

Os especialistas pontuaram pequenas sugestões de alteração nos itens. Embora todos os itens do instrumento tenham alcançado consenso quanto às equivalências, algumas sugestões foram consideradas para aprimorar a clareza. As sugestões foram analisadas e acatadas pelas pesquisadoras. Após a incorporação das sugestões dos especialistas e a realização das modificações necessárias nos itens, não foi necessário realizar demais rodadas de avaliação, pois as alterações foram apenas gramaticais e não interferiram no conteúdo do instrumento.

O pré-teste contou com a participação de trinta profissionais, sendo quinze enfermeiros e quinze técnicos de enfermagem, que atuavam na unidade de emergência referenciada e nas unidades de internação clínica/cirúrgica. A maioria dos profissionais tinha entre 31 e 40 anos (85%), era do sexo feminino (87%), e declarou mais de dez anos de experiência na instituição (84%). Todos os participantes consideraram o instrumento adequado para avaliar a percepção do cuidado centrado entre os profissionais, classificando os itens como compreensíveis ou parcialmente compreensíveis. O tempo médio de preenchimento do instrumento foi de 15 minutos, variando de 10 a 31 minutos.

Ao final da etapa do pré-teste, não foi observada sugestões, por parte dos participantes, para alteração dos itens.

Dos 307 profissionais que responderam ao instrumento, 240 (78%) foram mulheres e 67 (22%) homens. O tempo médio de experiência na área foi de 10 anos (85,1%). A faixa etária predominante foi de 41 e 50 anos (42,2%), enquanto 69,8% se autodeclararam brancos. O grau de escolaridade mais citado foi a especialização *lato sensu* (37,3%). Os resultados da validade convergente e confiabilidade do PCPI-S-VB estão apresentadas na Tabela 1.

**Tabela 1-:** Validade convergente do modelo fatorial e consistência interna da versão brasileira do *Person-centred Practice Inventory-Staff* (n = 307). Campinas, SP, Brasil, 2023

**Domínios**	AVE*	**Consistência Interna**		
		**Confiabilidade Composta**	**Alfa de Cronbach**	**Ômega de McDonald**
Profissionalmente competente	0,53	0,77	0,56	0,61
Habilidades interpessoais desenvolvidas	0,49	0,79	0,66	0,75
Estar comprometido com o trabalho	0,55	0,86	0,80	0,83
Conhecer a si mesmo	0,63	0,83	0,70	0,72
Clareza de crenças e valores	0,59	0,81	0,65	0,66
Combinação de habilidades	0,60	0,82	0,67	0,68
Tomada de decisão compartilhada	0,62	0,87	0,80	0,84
Relações de equipe eficazes	0,72	0,89	0,81	0,83
Compartilhamento de poder	0,61	0,86	0,79	0,81
Potencial para inovação e tomada de riscos	0,50	0,75	0,50	0,51
O ambiente físico	0,59	0,81	0,66	0,68
Sistemas organizacionais de apoio	0,63	0,89	0,85	0,88
Trabalhar com as crenças e valores do paciente	0,56	0,84	0,74	0,81
Tomada de decisão compartilhada	0,59	0,81	0,65	0,66
Engajamento	0,62	0,83	0,70	0,70
Ter presença solidária	0,71	0,88	0,79	0,80
Prestação de cuidados holísticos	0,71	0,88	0,79	0,81

*Variância Média Extraída

Os resultados da validade discriminante do modelo fatorial do PCPI-S-VB, pelo Critério de Fornell-Larcker estão apresentados na Tabela 2.

Na Tabela 3, estão apresentadas as Cargas Cruzadas do modelo fatorial do *Person-centred Practice Inventory-Staff*.

**Tabela 2-:** Validade Discriminante do modelo fatorial do *Person-centred Practice Inventory-Staff*, por meio do Critério de Fornell-Larcker* (n = 307). Campinas, SP, Brasil, 2023

**Domínio**	**Domínio**																
	**1.**	**2.**	**3.**	**4.**	**5.**	**6.**	**7.**	**8.**	**9.**	**10.**	**11.**	**12.**	**13.**	**14.**	**15.**	**16.**	**17.**
**1.**	**0,73**																
**2.**	0,49	**0,70**															
**3.**	0,41	0,59	**0,74**														
**4.**	0,28	0,41	0,42	**0,79**													
**5.**	0,41	0,49	0,52	0,44	**0,77**												
**6.**	0,30	0,49	0,48	0,36	0,51	**0,77**											
**7.**	0,39	0,30	0,44	0,34	0,49	0,40	**0,79**										
**8.**	0,32	0,29	0,34	0,29	0,43	0,21	0,49	**0,85**									
**9.**	0,38	0,33	0,40	0,30	0,50	0,32	0,57	0,69	**0,78**								
**10.**	0,53	0,43	0,50	0,39	0,49	0,45	0,56	0,49	0,64	**0,71**							
**11.**	0,59	0,49	0,49	0,41	0,46	0,47	0,41	0,39	0,46	0,54	**0,77**						
**12.**	0,32	0,34	0,36	0,32	0,42	0,32	0,56	0,66	0,71	0,56	0,49	**0,79**					
**13.**	0,41	0,47	0,60	0,39	0,50	0,45	0,39	0,41	0,45	0,57	0,60	0,47	**0,75**				
**14.**	0,45	0,39	0,47	0,32	0,44	0,37	0,39	0,33	0,38	0,49	0,54	0,39	0,71	**0,77**			
**15.**	0,38	0,53	0,54	0,41	0,45	0,55	0,35	0,33	0,40	0,50	0,57	0,37	0,67	0,65	**0,79**		
**16.**	0,30	0,49	0,55	0,34	0,41	0,48	0,28	0,35	0,35	0,45	0,51	0,37	0,64	0,61	0,65	**0,84**	
**17.**	0,37	0,51	0,56	0,39	0,42	0,48	0,25	0,28	0,27	0,47	0,51	0,30	0,66	0,56	0,63	0,73	**0,84**

Nota: 1. Profissionalmente competente; 2. Habilidades interpessoais desenvolvidas; 3. Estar comprometido com o trabalho; 4. Conhecer a si mesmo; 5. Clareza de crenças e valores; 6. Combinação de habilidades; 7. Tomada de decisão compartilhada; 8. Relações de equipe eficazes; 9. Compartilhamento de poder; 10. Potencial para inovação e tomada de riscos; 11. O ambiente físico; 12. Sistemas organizacionais de apoio; 13. Trabalhar com as crenças e valores do paciente; 14. Tomada de decisão compartilhada; 15. Engajamento; 16. Ter presença solidária; 17. Prestação de cuidados holísticos

*Valores da raiz quadrada de Variância Média Extraída em destaque

**Tabela 3-:** Validade Discriminante do Modelo fatorial do *Person-centred Practice Inventory-Staff*, por meio das Cargas Cruzadas (n = 307). Campinas, SP, Brasil, 2023

**Itens**	**1**	**2**	**3**	**4**	**5**	**6**	**7**	**8**	**9**	**10**	**11**	**12**	**13**	**14**	**15**	**16**	**17**
**1**	**0,61**	0,32	0,27	0,17	0,27	0,33	0,21	0,23	0,24	0,27	0,21	0,16	0,27	0,17	0,21	0,17	0,17
**2**	**0,80**	0,41	0,49	0,26	0,25	0,36	0,24	0,20	0,27	0,38	0,29	0,20	0,28	0,23	0,29	0,28	0,33
**3**	**0,77**	0,42	0,51	0,25	0,37	0,30	0,37	0,24	0,29	0,40	0,37	0,23	0,35	0,24	0,34	0,24	0,30
**4**	0,45	**0,65**	0,37	0,22	0,36	0,32	0,13	0,16	0,18	0,22	0,25	0,13	0,24	0,17	0,34	0,30	0,32
**5**	0,39	**0,73**	0,42	0,19	0,32	0,34	0,17	0,11	0,21	0,25	0,28	0,18	0,27	0,23	0,33	0,28	0,34
**6**	0,35	**0,77**	0,47	0,35	0,35	0,37	0,29	0,29	0,29	0,41	0,45	0,30	0,42	0,38	0,44	0,48	0,42
**7**	0,32	**0,65**	0,39	0,38	0,37	0,34	0,21	0,22	0,23	0,29	0,35	0,30	0,36	0,28	0,34	0,28	0,33
**8**	0,45	0,44	**0,67**	0,31	0,29	0,28	0,20	0,18	0,22	0,27	0,28	0,13	0,28	0,20	0,36	0,35	0,33
**9**	0,33	0,41	**0,74**	0,28	0,38	0,27	0,34	0,26	0,30	0,38	0,41	0,32	0,54	0,47	0,42	0,48	0,50
**10**	0,46	0,42	**0,74**	0,32	0,41	0,31	0,35	0,23	0,23	0,41	0,40	0,26	0,46	0,37	0,40	0,39	0,40
**11**	0,47	0,49	**0,79**	0,34	0,44	0,45	0,35	0,30	0,37	0,39	0,32	0,32	0,49	0,34	0,41	0,43	0,44
**12**	0,52	0,44	**0,77**	0,32	0,40	0,46	0,39	0,26	0,34	0,38	0,42	0,28	0,45	0,35	0,41	0,38	0,38
**13**	0,24	0,31	0,35	**0,83**	0,34	0,23	0,34	0,27	0,25	0,32	0,33	0,28	0,34	0,27	0,34	0,26	0,30
**14**	0,21	0,33	0,26	**0,78**	0,32	0,23	0,19	0,22	0,25	0,26	0,33	0,20	0,25	0,26	0,32	0,27	0,25
**15**	0,29	0,34	0,39	**0,76**	0,39	0,39	0,28	0,20	0,22	0,35	0,32	0,28	0,33	0,22	0,32	0,29	0,37
**16**	0,30	0,36	0,43	0,47	**0,75**	0,37	0,38	0,31	0,38	0,36	0,35	0,37	0,43	0,35	0,32	0,36	0,31
**17**	0,25	0,31	0,27	0,25	**0,74**	0,42	0,30	0,26	0,32	0,37	0,35	0,26	0,31	0,29	0,30	0,25	0,30
**18**	0,37	0,45	0,47	0,30	**0,81**	0,39	0,43	0,41	0,45	0,41	0,36	0,33	0,41	0,38	0,41	0,32	0,34
**19**	0,35	0,33	0,34	0,23	0,28	**0,76**	0,23	0,05	0,15	0,26	0,33	0,16	0,32	0,23	0,39	0,30	0,33
**20**	0,35	0,37	0,40	0,29	0,54	**0,83**	0,39	0,25	0,34	0,43	0,41	0,34	0,42	0,33	0,43	0,39	0,39
**21**	0,34	0,44	0,37	0,32	0,33	**0,73**	0,30	0,16	0,23	0,33	0,34	0,23	0,31	0,29	0,46	0,41	0,39
**22**	0,36	0,32	0,40	0,27	0,47	0,45	**0,78**	0,36	0,40	0,43	0,33	0,32	0,33	0,35	0,37	0,24	0,22
**23**	0,26	0,27	0,37	0,35	0,37	0,31	**0,83**	0,38	0,41	0,43	0,35	0,46	0,30	0,29	0,24	0,23	0,21
**24**	0,27	0,20	0,35	0,26	0,38	0,27	**0,80**	0,45	0,50	0,45	0,33	0,55	0,32	0,30	0,25	0,21	0,19
**25**	0,32	0,13	0,27	0,20	0,31	0,23	**0,76**	0,37	0,48	0,47	0,28	0,44	0,29	0,30	0,23	0,19	0,17
**26**	0,33	0,31	0,35	0,28	0,42	0,27	0,45	**0,90**	0,62	0,48	0,33	0,58	0,38	0,29	0,32	0,34	0,28
**27**	0,27	0,27	0,29	0,24	0,36	0,18	0,43	**0,91**	0,63	0,44	0,36	0,60	0,36	0,27	0,29	0,31	0,24
**28**	0,14	0,13	0,20	0,23	0,31	0,06	0,36	**0,74**	0,51	0,31	0,31	0,50	0,32	0,30	0,22	0,25	0,17
**29**	0,25	0,23	0,30	0,16	0,38	0,18	0,35	0,60	**0,78**	0,43	0,34	0,57	0,39	0,29	0,31	0,27	0,19
**30**	0,37	0,27	0,40	0,28	0,46	0,31	0,44	0,41	**0,73**	0,52	0,39	0,42	0,40	0,39	0,44	0,35	0,28
**31**	0,23	0,18	0,22	0,19	0,33	0,23	0,42	0,55	**0,77**	0,47	0,30	0,58	0,23	0,18	0,21	0,18	0,13
**32**	0,29	0,24	0,31	0,29	0,38	0,27	0,54	0,60	**0,83**	0,55	0,39	0,67	0,35	0,29	0,27	0,27	0,22
**33**	0,33	0,25	0,31	0,23	0,36	0,18	0,55	0,57	**0,69**	0,72	0,34	0,62	0,36	0,28	0,30	0,20	0,20
**34**	0,36	0,36	0,33	0,31	0,34	0,44	0,38	0,21	**0,36**	0,72	0,40	0,37	0,43	0,37	0,35	0,38	0,39
**35**	0,34	0,30	0,42	0,30	0,34	0,33	0,26	0,24	**0,30**	0,68	0,41	0,19	0,42	0,41	0,43	0,39	0,42
**36**	0,34	0,34	0,36	0,36	0,23	0,34	0,21	0,23	**0,31**	0,36	0,70	0,24	0,41	0,43	0,44	0,35	0,36
**37**	0,27	0,36	0,37	0,29	0,38	0,38	0,25	0,32	**0,34**	0,40	0,81	0,4	0,47	0,42	0,45	0,41	0,40
**38**	0,33	0,42	0,41	0,31	0,43	0,37	0,46	0,34	**0,40**	0,48	0,80	0,47	0,49	0,40	0,44	0,42	0,42
**39**	0,17	0,19	0,21	0,31	0,28	0,22	0,41	0,50	**0,49**	0,34	0,35	0,71	0,29	0,23	0,23	0,20	0,11
**40**	0,14	0,30	0,23	0,20	0,30	0,18	0,37	0,45	**0,47**	0,33	0,36	0,74	0,29	0,28	0,22	0,23	0,19
**41**	0,25	0,28	0,29	0,30	0,34	0,30	0,46	0,55	**0,57**	0,48	0,4	0,82	0,38	0,34	0,31	0,32	0,25
**42**	0,26	0,3	0,34	0,23	0,37	0,29	0,43	0,54	**0,66**	0,51	0,44	0,83	0,45	0,36	0,36	0,38	0,32
**43**	0,25	0,25	0,31	0,25	0,37	0,27	0,54	0,56	**0,60**	0,51	0,38	0,84	0,44	0,33	0,31	0,29	0,28
**44**	0,35	0,37	0,39	0,29	0,25	0,40	0,24	0,30	**0,33**	0,41	0,38	0,33	0,66	0,45	0,47	0,40	0,42
**45**	0,28	0,34	0,46	0,29	0,33	0,32	0,21	0,26	**0,29**	0,38	0,40	0,29	0,81	0,59	0,56	0,54	0,60
**46**	0,32	0,35	0,51	0,27	0,49	0,31	0,38	0,35	**0,33**	0,49	0,46	0,41	0,76	0,56	0,46	0,54	0,48
**47**	0,30	0,36	0,45	0,32	0,42	0,34	0,33	0,33	**0,39**	0,43	0,55	0,38	0,76	0,52	0,51	0,44	0,48
**48**	0,20	0,31	0,36	0,19	0,36	0,27	0,27	0,27	**0,25**	0,40	0,38	0,26	0,59	0,80	0,53	0,46	0,51
**49**	0,22	0,30	0,42	0,24	0,40	0,30	0,42	0,29	**0,33**	0,39	0,42	0,40	0,55	0,79	0,44	0,45	0,39
**50**	0,26	0,28	0,29	0,31	0,25	0,29	0,19	0,19	**0,29**	0,34	0,44	0,23	0,48	0,71	0,52	0,50	0,40
**51**	0,35	0,46	0,46	0,37	0,31	0,49	0,28	0,24	**0,26**	0,39	0,50	0,25	0,52	0,47	0,79	0,48	0,55
**52**	0,27	0,38	0,35	0,32	0,39	0,33	0,26	0,28	**0,34**	0,42	0,37	0,30	0,49	0,48	0,76	0,45	0,43
**53**	0,30	0,41	0,46	0,29	0,37	0,47	0,28	0,26	**0,35**	0,39	0,48	0,33	0,57	0,58	0,82	0,60	0,52
**54**	0,23	0,42	0,44	0,25	0,34	0,43	0,24	0,34	**0,33**	0,34	0,45	0,34	0,55	0,59	0,64	0,88	0,60
**55**	0,26	0,39	0,47	0,33	0,36	0,39	0,27	0,28	**0,33**	0,40	0,45	0,33	0,61	0,52	0,52	0,86	0,64
**56**	0,33	0,42	0,47	0,29	0,33	0,39	0,18	0,27	**0,22**	0,41	0,39	0,26	0,46	0,42	0,48	0,79	0,60
**57**	0,33	0,37	0,38	0,32	0,29	0,42	0,20	0,16	**0,15**	0,35	0,36	0,14	0,47	0,36	0,44	0,53	0,75
**58**	0,29	0,43	0,48	0,29	0,36	0,38	0,22	0,24	**0,21**	0,41	0,44	0,28	0,58	0,55	0,56	0,64	0,87
**59**	0,34	0,47	0,53	0,37	0,38	0,42	0,21	0,28	**0,30**	0,43	0,48	0,31	0,59	0,49	0,58	0,66	0,90

## Discussão

O processo de adaptação transcultural do PCPI-S-versão brasileira seguiu as etapas recomendadas internacionalmente^(15-19)^ sem grandes desafios, devido ao compromisso do comitê na avaliação dos 59 itens, bem como à estrutura objetiva e ao arcabouço teórico do instrumento^(6-7)^.

Partindo do pressuposto de que a leitura, a interpretação e o consequente preenchimento de um instrumento geram não apenas dados, mas instigam os sujeitos ao exercício de reflexões sobre a sua prática e um posterior movimento de mudança visando o seu aprimoramento ou ajustes na performance, atentou-se, durante todo o processo de tradução e adaptação do instrumento, para replicar o construto original^(15)^.

Com o objetivo de estimular a autoanálise e a sensibilização dos sujeitos, colocando-se na situação apresentada pela declaração, foram readequadas todas as afirmações iniciando as afirmações, com o pronome “eu” e substituídos os termos “levo” e “uso”, para os termos “dedico” e “costumo”, nos itens 13 e 14, respectivamente.

Na análise do parecer dos especialistas, o termo “*challenge”*, presente nos itens 7 e 17, gerou ambiguidades, sendo interpretado por alguns de forma pejorativa, associando-o a competição e conflitos interpessoais – contrários ao modelo de cuidado proposto. Considerando que a declaração visa investigar o posicionamento do sujeito em relação à equipe diante de divergências de práticas, optou-se, após consenso entre pesquisadoras, comitê de especialistas e linguista, pela substituição do termo por”confronto”. O termo *“ward rounds*”, presente no item 25, foi traduzido como “rodadas de enfermagem”, expressão frequentemente utilizada em países europeus para descrever a passagem de plantão beira leito realizada pelos profissionais da enfermagem na troca dos turnos. No entretanto, essa terminologia não é comum na cultura brasileira, o que poderia gerar interpretações divergentes entre os participantes. Diante disso, optou-se por readequar o termo para “passagem de plantão beira-leito”, visando facilitar a compreensão da declaração.

A validação de conteúdo do PCPI-S, reconhecida internacionalmente como a etapa mais relevante no processo de validação de instrumentos^(16)^, contou com a ampla experiência e formação das participantes do comitê de especialistas. Foram analisadas as avaliações de 15 profissionais atuantes em gestão, ensino e pesquisa. Como resultado, a versão do PCPI-P obteve IVC superior a 0,87 e RVC superior a 0,73 para todos os itens, superando os valores recomendados para análise por 15 juízes^(16)^.

Após finalizada as adequações sugeridas pelo comitê de especialistas, elaborou-se a versão pré-final do PCPI-S, que, posteriormente, foi submetida ao pré-teste. O tempo médio de resposta ao instrumento foi de 15 minutos, de forma que os participantes consideraram o instrumento adequado e compreensível.

Após finalização do pré-teste, seguiu-se com a etapa de avaliação das propriedades de medida do instrumento, que correspondeu à avaliação da validade de construto convergente e discriminante e, confiabilidade do PCPI-S.

Na análise fatorial confirmatória os valores da AVE e das cargas cruzadas foram superiores a 0,5, confirmando a validade de constructo convergente e manteve-se 17 domínios do PCPI-S original^(17-18)^. Embora apenas o domínio 2 “Habilidades interpessoais desenvolvidas” tenha resultado em 0,49 de AVE, muito próximo de 0,5 para sugerir validade convergente, todos os itens do domínio apresentaram carga fatorial superior a 0,65 indicando evidência de validade convergente também neste domínio^(17-18)^.

A análise também apresenta evidências de validade discriminante, com base no Critério de Fornell-Larcker, no qual se verifica que os valores de correlação dentro de cada domínio foram superiores às correlações entre os domínios distintos^(17-18)^. No que se refere à confiabilidade, a análise da consistência interna do instrumento indica que, embora o alfa de Cronbach tenha apresentado valores entre 0,5 e 0,8, esse coeficiente é uma estimativa da confiabilidade para medir um único construto comum a todos os itens de um domínio, desde que este seja unidimensional e apresente cargas fatoriais semelhantes. Como os itens dentro do mesmo domínio apresentaram comportamento multifatorial, foram realizadas medidas alternativas de confiabilidade, como o Ômega de McDonald, que que variou entre 0,61 e 0,88, e a avaliação da confiabilidade composta, que não assume unidimensionalidade dos itens, sendo, portanto, uma medida mais imparcial. A confiabilidade composta apresentou valores entre 0,77 e 0,89, indicando evidências de consistência interna do PCPI-S-VB e reforçando a precisão do instrumento^(15,17)^.

Diferentemente da tradução malaia^(10)^, que enfrentou desafios na tradução e adaptação, devido à preocupação em manter a interdisciplinaridade do instrumento, além de ter sido realizada em um contexto de cuidados primários com uma amostra multiprofissional, o processo de adaptação transcultural para o cenário brasileiro não apresentou dificuldades significativas. Na versão malaia, a utilização da análise fatorial exploratória resultou na exclusão de 6 dos 17 domínios. Já na adaptação para o Brasil, foi possível manter a estrutura original do instrumento, em consonância com as demais traduções^(7-9,11-14)^.

Conceitualmente, o referencial teórico da prática centrada na pessoa defende que a percepção de todas as pessoas envolvidas no processo do cuidado, deve ser considerada e valorizada, incentivando o exercício de relações harmoniosas, consequentemente fortalecendo o vínculo entre profissionais, familiares e pacientes^(6)^. Assim, a utilização do PCPI-S-VB pode sensibilizar os profissionais de saúde para dimensões não técnicas do cuidado, como a experiência do paciente^(20-23)^. Nessa perspectiva, acredita-se que o uso do instrumento validado neste estudo favoreça, entre os profissionais, um ciclo virtuoso de cuidado, integrando as dimensões afetiva, cognitiva, comportamental, moral e empática do cuidado centrado na pessoa. A interação entre essas dimensões possibilita que os profissionais compartilhem e compreendam os sentimentos da pessoa doente, aprimorando a comunicação e promovendo um cuidado mais humanizado. Dessa forma, o desejo de ajudar passa a ser impulsionado por uma motivação interna genuína dos profissionais de saúde^(23)^.

Sendo assim, a principal contribuição deste estudo é a disponibilização da versão brasileira do *Person-centred Practice Inventory-Staff* para sensibilizar profissionais de saúde e promover o cuidado centrado na pessoa e sua família. A validação do instrumento em outros países^(8-14)^ indica sua credibilidade, possibilitando a comparabilidade de dados e a identificação de estratégias para desenvolvimento de profissionais com competências clínicas e habilidades comportamentais voltadas para ao cuidado centrado na pessoa.

O PCPI-S original foi desenvolvido para ser aplicado em diferentes ambientes de cuidado e direcionado à equipe multiprofissional da saúde. Dessa forma, a principal limitação deste estudo é que a coleta de dados para análise das propriedades de medida da versão brasileira do PCPI-S foi realizada apenas com profissionais de enfermagem, em uma única instituição hospitalar. Portanto, recomenda-se a aplicação da versão brasileira do PCPI-S em diferentes cenários de cuidado, envolvendo outros profissionais da equipe multiprofissional.

## Conclusão

A versão brasileira do *Person-centred Practice Inventory-Staff* – versão brasileira demonstrou evidências de validade e consistência interna para medir a percepção da prática do cuidado centrado na pessoa entre profissionais da enfermagem no âmbito hospitalar.

Reitera-se a importância de investigar e validar a percepção das demais categorias profissionais frente ao cuidado centrado na pessoa em um cenário amostral mais amplo. Considerando que o instrumento pode ser aplicado a diferentes categorias profissionais e em diversos contextos de cuidado, torna-se relevante explorar populações e ambientes diversos.

## Data Availability

Todos os dados gerados ou analisados durante este estudo estão incluídos neste artigo publicado.

# Translation: Adaptación transcultural y validación del *Person-centred Practice Inventory-Staff* para la cultura brasileña*

**DOI:** 10.1590/1518-8345.7803.4664

**Authors:** Juliana Andrioli Nucci, Edinêis de Brito Guirardello, Ariane Polidoro Dini

**Affiliations:** 1 Universidade Estadual de Campinas, Faculdade de Enfermagem, Campinas, SP, Brasil

**Keywords:** Proceso de Traducción, Estudios Transculturales, Estudios de Validación, Atención Dirigida al Paciente, Psicometría, Reproducibilidad de los Resultados

## Abstract

realizar la traducción, adaptación transcultural y validación del *Person-centred Practice Inventory-Staff* al portugués de Brasil.

estudio metodológico que siguió las etapas de traducción, síntesis, retrotraducción, evaluación por especialistas y prueba piloto. La validez de contenido fue evaluada mediante el Índice de Validez de Contenido y la Razón de Validez de Contenido. La validez de constructo convergente y discriminante fue verificada mediante el análisis factorial confirmatorio. La consistencia interna fue verificada por el alfa de Cronbach, la Fiabilidad Compuesta y el Omega de McDonald.

participaron 15 profesionales en la validación de contenido y 307 en la evaluación del constructo y fiabilidad. La validez de contenido obtuvo valores superiores a 0,87 para el Índice de Validez de Contenido y 0,73 para la Razón de Validez de Contenido. El análisis factorial confirmatorio distribuyó los 59 ítems en los 17 dominios originales, con varianza media extraída superior a 0,49 y cargas factoriales superiores a 0,61. La consistencia interna presentó alfa de Cronbach entre 0,56 y 0,85; fiabilidad compuesta entre 0,75 y 0,89; y Omega de McDonald entre 0,61 y 0,88.

la versión brasileña del *Person-centred Practice Inventory-Staff* demostró validez y fiabilidad satisfactorias para evaluar la percepción de la práctica del cuidado centrado en la persona entre profesionales de enfermería en el contexto hospitalario.

## Destacados

**(1)** El instrumento PCPI-S-VB permite la sensibilización respecto al cuidado centrado en la persona. **(2)** El PCPI-S-VB validado en países europeos permitirá la comparabilidad internacional. **(3)** La versión brasileña del PCPI-S-VB presenta evidencias de validez y confiabilidad. **(4)** El instrumento PCPI-S-VB contribuye a la prestación de un cuidado más seguro.

## Introducción

En busca de atribuir excelencia a los estándares de seguridad y calidad asistencial, gestores y políticos de salud han buscado nuevas estrategias para sensibilizar a los profesionales responsables del cuidado, incentivando la adopción de un enfoque singular y humanizado^(1)^.

El cuidado centrado en la persona (CCP) se construye a partir de la valorización de la percepción y expectativas no solo de la persona que recibe el cuidado, sino de todos los involucrados, es decir, familiares, cuidadores y profesionales de la salud^(2)^. El CCP es una práctica de cuidado establecida mediante la formación y promoción de relaciones saludables entre cuidadores, usuarios de los servicios y personas significativas en sus vidas. Está sustentado en valores de respeto por las personas, derecho individual a la autodeterminación, respeto mutuo y comprensión, siendo posibilitado por culturas de empoderamiento que promueven enfoques continuos para el desarrollo de la práctica^(3)^.

A pesar de la relevancia del CCP, su adhesión enfrenta desafíos a nivel global, principalmente por confrontarse con el modelo biomédico convencional, factores culturales ligados a las organizaciones, el entorno del cuidado, el perfil de los profesionales, además de la carencia de instrumentos confiables que ayuden en el monitoreo y evaluación de la práctica en los servicios^(2,4-5)^. En esta perspectiva, la atención de la comunidad científica se ha centrado en explorar la fundamentación teórica del CCP, con el objetivo de definir de forma consensuada y precisa los conceptos y definiciones^(2)^.

El marco teórico de McCormack y McCance presenta una estructura teórica para el CCP, conocida como *Person-centred Practice Framework* (PCPF). Esta es descrita como una teoría de alcance medio sustentada por cuatro constructos: (1) prerrequisitos, que abordan las características del profesional; (2) el entorno del cuidado, que se refiere al contexto en el que se presta el cuidado; (3) procesos de cuidado, que se centran en la forma en que se brinda el cuidado; y (4) resultados centrados en la persona, que se enfocan en lograr resultados eficaces centrados en la persona. De acuerdo con la teoría, los atributos del profesional se consideran imprescindibles para la gestión de un entorno de cuidado capaz de proporcionar con eficacia resultados centrados en la persona^(6)^.

Con el objetivo de investigar cómo perciben las perspectivas del CCP los equipos y usuarios de los servicios de salud, se desarrolló el *Person-centred Practice Inventory-Staff* (PCPI-S). Este instrumento propone ofrecer, desde la perspectiva de los profesionales, aportes a las organizaciones y gestores para la elaboración de un plan de cuidados que tenga a la persona como foco central^(7)^.

El PCPI-S tiene como finalidad analizar la percepción de los profesionales sobre la práctica centrada en la persona^(7)^. Este instrumento abarca 59 ítems distribuidos en 17 dominios que permiten la autoevaluación del equipo multiprofesional en relación con su práctica centrada en la persona, y ha sido validado para su aplicación en diversos contextos asistenciales. Se trata de un instrumento que presentó validez de contenido, de constructo y consistencia interna en Noruega, Austria, Malasia, Corea, Alemania y Portugal^(7-14)^.

En Brasil, la evaluación del CCP es incipiente y carece de instrumentos que posibiliten la evaluación de la percepción de los profesionales en relación con esta práctica. El objetivo fue realizar la traducción, adaptación transcultural y validación del *Person-centred Practice Inventory-Staff* al portugués de Brasil.

## Método

### Diseño del estudio

Estudio metodológico que siguió las siguientes etapas: traducción, síntesis de las traducciones, retrotraducción, análisis por un comité de expertos, prueba de la versión final y envío al autor del instrumento original. De forma secuencial, se analizaron la validez de constructo y la consistencia interna del PCPI-S^(15-18)^.

### Período

El período de recolección de datos tuvo lugar después de la aprobación del estudio por el Comité de Ética en Investigación, entre octubre de 2022 y diciembre de 2023.

### Traducción y adaptación transcultural

La primera etapa consistió en la traducción de la versión original del instrumento del inglés al portugués de Brasil por dos traductores independientes, con experiencia y fluidez en ambos idiomas, cuya lengua materna era el portugués. Solo uno de los traductores fue informado sobre los conceptos y objetivos del PCPI-S, de manera que el desconocimiento de esta información permitiera que el segundo traductor estuviera más sensible para detectar diferentes significados del instrumento original^(15)^.

Las dos versiones traducidas fueron denominadas T1 y T2, y se compararon con el instrumento original y se sintetizaron, generando una versión única, denominada versión síntesis de las traducciones (T12)^(15)^. Esta etapa fue desarrollada por un tercer traductor independiente, cuya lengua materna era el portugués y tenía fluidez en inglés^(15)^.

La etapa de retrotraducción comprendió la traducción de la versión síntesis (T12) de vuelta al idioma original, generando dos versiones (RT1 y RT2). Esta etapa fue realizada por dos traductores independientes cuya lengua materna correspondía a la del instrumento original (inglés). En esta fase, los profesionales no fueron informados sobre los objetivos del PCPI-S^(15)^.

Finalizadas las etapas de traducción, síntesis y retrotraducción, se procedió a la evaluación de todas las versiones (T1, T2, T12, RT1 y RT2) por parte del comité de expertos para desarrollar la versión prefinal del PCPI-S^(15)^. Para la composición del comité de expertos, los miembros fueron invitados a partir del análisis curricular en la *Plataforma Lattes*, siendo seleccionados de forma intencional profesionales con experiencia en la traducción y adaptación de instrumentos y con participación en investigaciones relacionadas con el cuidado centrado en la persona, la gestión de seguridad y la calidad asistencial. La invitación fue enviada por correo electrónico. Tras la aceptación, se proporcionó el enlace de acceso al instrumento y al formulario de análisis de equivalencias.

El proceso de análisis de las versiones traducidas y retrotraducidas del PCPI-S por el comité de expertos permitió la comparación de las traducciones iniciales (T1 y T2), la versión síntesis (T12) y las retrotraducciones (RT1 y RT2), y posibilitó examinar las equivalencias semántica, idiomática, cultural y conceptual, además de su validez de contenido^(15)^.

La validez de contenido del PCPI-S fue analizada mediante el Índice de Validez de Contenido (IVC) y la Razón de Validez de Contenido (RVC)^(16-17)^.

Para el cálculo del IVC, se consideró una escala ordinal de cuatro puntos, con las opciones: (1) No relevante o no presenta claridad; (2) Requiere una revisión extensa para ser relevante o presentar claridad; (3) Requiere una pequeña revisión para ser relevante o presentar claridad; o (4) Relevante y representativo. El cálculo del IVC se realiza mediante la suma del número de respuestas “3” y “4” dividida por el número total de encuestados^(16)^.

Para la evaluación de la relevancia de los ítems del PCPI-S, se utilizó la RVC, en la cual los expertos evaluaron los ítems del instrumento a partir de una escala ordinal de tres puntos, siendo: 1) Ítem innecesario; 2) Ítem útil, pero innecesario; 3) Ítem esencial^(16)^.

Para la validez de contenido, considerando el número de participantes en la evaluación, se consideraron valores aceptables: IVC superior a 0,80 y RVC superior a 0,54^(16)^.

Tras el análisis de todas las versiones traducidas de los ítems del PCPI-S y las sugerencias del comité, se obtuvo la versión prefinal del PCPI-S para la realización del procedimiento de prueba piloto^(15)^. La etapa de prueba piloto comprendió la última fase del proceso de traducción y adaptación transcultural y consistió en la aplicación de la versión prefinal del instrumento en una muestra de 30 sujetos, profesionales del área asistencial, entre ellos técnicos en enfermería y enfermeros. Los profesionales fueron seleccionados intencionalmente mediante la indicación de los supervisores de área de los sectores correspondientes al campo de estudio. La invitación para participar en la investigación fue enviada por intermedio de correo electrónico y una aplicación de mensajería instantánea, proporcionados por los supervisores. Tras aceptar participar en la investigación, los profesionales tuvieron acceso al enlace del Consentimiento Informado, que fue completado antes de acceder al formulario correspondiente al contenido de la etapa de prueba piloto. Los profesionales fueron orientados a evaluar las afirmaciones del instrumento en cuanto a la facilidad de comprensión y claridad del contenido, registrando, cuando fuera necesario, sugerencias de mejora para el perfeccionamiento del contenido.

Tras la etapa de prueba piloto, se envió un correo electrónico a los autores del PCPI-S con todas las versiones del proceso de adaptación transcultural, así como la versión brasileña del instrumento.

### Análisis de las propiedades de medida del PCPI-S versión brasileña

El PCPI-S fue desarrollado a partir de la estructura teórica del PCP-F, que aborda la relación terapéutica entre los profesionales, la persona a ser cuidada, sus familiares y cuidadores^(6-7)^.

El instrumento cuenta con 59 ítems, distribuidos en 17 dominios guiados por tres constructos^(7)^. Los dominios relacionados con los prerrequisitos profesionales incluyen la competencia profesional (ítems 1, 2 y 3), el desarrollo de habilidades interpersonales (ítems 4, 5, 6 y 7), el compromiso profesional con el trabajo (ítems 8, 9, 10, 11 y 12), el autoconocimiento (ítems 13, 14 y 15) y la claridad de creencias y valores (ítems 14, 15, 16, 17 y 18)^(7)^. Los aspectos relacionados con el dominio del entorno del cuidado incluyen la capacidad de combinar habilidades (ítems 19, 20 y 21), el ejercicio de compartir la toma de decisiones (ítems 22, 23, 24 y 25), el establecimiento de relaciones interprofesionales eficaces (ítems 26, 27 y 28), el reparto de poder (ítems 29, 30, 31 y 32), el potencial para la innovación y la toma de riesgos (ítems 33, 34 y 35), las características del entorno físico (ítems 36, 37 y 38), los sistemas organizacionales de apoyo (ítems 39, 40, 41, 42 y 43) y el manejo de creencias y valores (ítems 44, 45, 46 y 47)^(7)^. Los dominios que abordan los procesos centrados en la persona implican la toma de decisiones compartida (ítems 48, 49 y 50), estrategias de involucramiento de la persona (ítems 51, 52 y 53), actitud solidaria (ítems 54, 55 y 56) y prestación de cuidados holísticos (ítems 57, 58 y 59)^(7)^.

Las respuestas a los ítems se realizan mediante una escala ordinal de cinco puntos, cuyas opciones son: totalmente en desacuerdo (1), en desacuerdo (2), neutral (3), de acuerdo (4) y totalmente de acuerdo (5)^(7)^.

La puntuación se obtiene mediante el cálculo del puntaje promedio de cada dominio, sumando la puntuación de todos los ítems correspondientes al dominio y dividiendo el total por el número de ítems del dominio^(7)^. Un promedio alto del porcentaje de respuestas indica una percepción positiva de la práctica en el entorno del cuidado^(7)^.

El análisis de la validez de constructo y la confiabilidad de la versión brasileña del PCPI-S se obtuvo mediante su aplicación en una muestra de 307 profesionales de enfermería de un hospital universitario de gran porte.

El lugar de recolección de los datos fue un hospital general, de enseñanza, en el interior del estado de São Paulo, con capacidad física para 403 camas distribuidas entre diferentes especialidades y que ofrece atención especializada de alta complejidad, financiada por el Sistema Único de Salud (SUS).

Para la recolección y gestión de los datos se utilizó el formulario *Google Forms*® y la hoja de cálculo del programa *Excel*® para el almacenamiento de la base de datos.

Se consideraron elegibles para el estudio todos los enfermeros y técnicos de enfermería con vínculo laboral en la institución por un período mínimo de seis meses. Los criterios de exclusión estuvieron relacionados con situaciones de ausencias, vacaciones o licencias médicas durante el período de recolección de datos.

La invitación para participar en la investigación fue enviada por correo electrónico y una aplicación de mensajería instantánea, proporcionados por los supervisores. Tras aceptar participar en la investigación, los profesionales accedieron al enlace del Consentimiento Informado, que fue completado antes de acceder al formulario *Google Forms*®.

### Tratamiento y análisis de los datos

El análisis descriptivo se realizó a partir del levantamiento de las características sociodemográficas y profesionales de los sujetos participantes de la investigación. Los porcentajes de las variables fueron calculados a partir de los datos recolectados en la plataforma *Google Forms*®.

La validez de constructo se llevó a cabo mediante el análisis factorial confirmatorio de segundo orden. Se utilizaron modelos de ecuaciones estructurales considerando como método de estimación el *Partial Least Squares* (PLS), o mínimos cuadrados parciales. Para la implementación de estos análisis se utilizó el *softwareSmart* PLS 3.3.5.

Para la evaluación de la validez de constructo, en primer lugar se evaluó la validez convergente del modelo, mediante el *Average Variance Extracted* (AVE) para cada uno de los 17 dominios del instrumento. Esta medida evalúa la proporción de la varianza de los ítems que es explicada por el factor/dominio al que pertenecen. Para que la validez convergente sea adecuada, los valores de AVE deben ser superiores a 0,5^(17-18)^.

La validez discriminante fue evaluada mediante el criterio de Fornell-Larcker^(17-18)^, que compara si la raíz cuadrada del AVE para determinado dominio es superior a los valores de correlación entre los demás dominios^(17-18)^. Otro criterio considerado para evaluar la validez discriminante fue el análisis de cargas cruzadas. En este caso, se verificó si la carga factorial de un ítem era más elevada en el dominio al cual fue originalmente asignado^(17-18)^, es decir, si cada uno de los 59 ítems del PCPI-S presentaba una carga factorial superior a 0,5 dentro de su respectivo dominio^(17-18)^.

La confiabilidad fue evaluada con base en la consistencia interna. Para ello, se consideraron el alfa de Cronbach, la fiabilidad compuesta y el omega de McDonald, siendo que valores iguales o superiores a 0,6 indican una consistencia satisfactoria^(17-18)^.

### Aspectos éticos

El estudio fue aprobado por el Comité de Ética en Investigación de la *Universidade Estadual de Campinas* (UNICAMP) [Certificado de Presentación para Apreciación Ética (CAAE): 62881722.6.0000.5404] y se obtuvo el consentimiento de todos los sujetos mediante la firma del Consentimiento Informado, conforme a la Resolución del Consejo Nacional de Salud/Ministerio de Salud (CNS/MS) 466/12.

## Resultados

El proceso de traducción y adaptación del instrumento se llevó a cabo sin dificultades, cumpliendo con las recomendaciones internacionales. Para la composición del comité de expertos, se envió la invitación a aproximadamente veintidós profesionales por correo electrónico, obteniéndose la devolución del formulario completado por quince profesionales.

El comité estuvo compuesto exclusivamente por mujeres, entre ellas una enfermera docente con experiencia en la traducción y adaptación de instrumentos y experiencia en el campo asistencial en servicios de salud pertenecientes a la cultura de origen del instrumento; nueve enfermeras docentes con experiencia en la gestión del cuidado centrado en la persona, seguridad y calidad asistencial; una enfermera doctoranda involucrada en estudios relacionados con la seguridad del paciente; dos lingüistas con experiencia en el proceso de traducción y adaptación de instrumentos; una médica especialista en gestión de calidad y seguridad del paciente; y una enfermera docente e investigadora del cuidado centrado en la persona. Se realizó una ronda de análisis por parte del comité de expertos antes de la etapa de prueba piloto. En esta etapa, se calcularon los valores de IVC y RVC para todos los ítems del instrumento.

En la evaluación de la validez de contenido, los ítems 3, 5, 7, 8, 9, 11, 12, 18, 23, 28, 30, 31, 33, 35, 36, 38, 39, 40, 43, 44, 45, 46, 47, 48, 49, 50, 51, 55, 58 y 59 presentaron un IVC de 1,0. Presentaron un IVC de 0,93 los ítems: 1, 2, 4, 6, 10, 13, 15, 16, 17, 19, 21, 22, 24, 25, 26, 27, 29, 41, 42, 53, 54, 57. Presentaron un IVC de 0,87 los ítems: 14, 20, 32, 34, 37, 52, 56.

Con respecto a la RVC, presentaron un valor de 1,0 los ítems 1, 3, 8, 9, 10, 11, 12, 18, 23, 25, 28, 31, 37, 40, 41, 42, 43, 46, 47, 49, 50, 52, 54, 55, 58. Presentaron una RVC de 0,87 los ítems: 2, 4, 5, 6, 7, 15, 16, 17, 19, 21, 22, 24, 26, 27, 29, 30, 32, 35, 36, 38, 39, 52, 53, 56, 57. Presentaron una RVC de 0,73 los ítems 13, 14, 20, 33, 34 y 53.

Los expertos señalaron pequeñas sugerencias de modificación en los ítems. Aunque todos los ítems del instrumento alcanzaron consenso en cuanto a las equivalencias, algunas sugerencias fueron consideradas para mejorar la claridad. Estas sugerencias fueron analizadas y aceptadas por las investigadoras. Tras la incorporación de las sugerencias de los expertos y la realización de las modificaciones necesarias en los ítems, no fue necesario realizar nuevas rondas de evaluación, ya que las alteraciones fueron únicamente gramaticales y no afectaron el contenido del instrumento.

La prueba piloto contó con la participación de treinta profesionales, siendo quince enfermeros y quince técnicos en enfermería, que trabajaban en la unidad de emergencia referenciada y en las unidades de hospitalización clínica/quirúrgica. La mayoría de los profesionales tenía entre 31 y 40 años (85%), era del sexo femenino (87%) y declaró más de diez años de experiencia en la institución (84%). Todos los participantes consideraron el instrumento adecuado para evaluar la percepción del cuidado centrado entre los profesionales, clasificando los ítems como comprensibles o parcialmente comprensibles. El tiempo medio de llenado del instrumento fue de 15 minutos, con un rango de 10 a 31 minutos.

Al final de la etapa de prueba piloto, no se observaron sugerencias por parte de los participantes para modificar los ítems.

De los 307 profesionales que respondieron al instrumento, 240 (78%) eran mujeres y 67 (22%) hombres. El tiempo medio de experiencia en el área fue de 10 años (85,1%). El rango de edad predominante fue de 41 a 50 años (42,2%), mientras que el 69,8% se autodeclaró blanco. El nivel educativo más mencionado fue la especialización *lato sensu* (37,3%). Los resultados de la validez convergente y confiabilidad del PCPI-S-VB se presentan en la Tabla 1.

**Tabla 1-:** Validez convergente del modelo factorial y consistencia interna de la versión brasileña del *Person-centred Practice Inventory-Staff* (n = 307). Campinas, SP, Brasil, 2023

**Dominios**	AVE*	**Consistencia Interna**		
		**Fiabilidad Compuesta**	**Alfa de Cronbach**	**Omega de McDonald**
Profesionalmente competente	0,53	0,77	0,56	0,61
Habilidades interpersonales desarrolladas	0,49	0,79	0,66	0,75
Estar comprometido con el trabajo	0,55	0,86	0,80	0,83
Conocer a si mismo	0,63	0,83	0,70	0,72
Claridad de creencias y valores	0,59	0,81	0,65	0,66
Combinación de habilidades	0,60	0,82	0,67	0,68
Toma de decisiones compartida	0,62	0,87	0,80	0,84
Relaciones de equipo eficaces	0,72	0,89	0,81	0,83
Distribución de poder	0,61	0,86	0,79	0,81
Potencial para la innovación y toma de riesgos	0,50	0,75	0,50	0,51
El entorno físico	0,59	0,81	0,66	0,68
Sistemas organizacionales de apoyo	0,63	0,89	0,85	0,88
Trabajar con las creencias y valores del paciente	0,56	0,84	0,74	0,81
Toma de decisiones compartida	0,59	0,81	0,65	0,66
Compromiso	0,62	0,83	0,70	0,70
Tener presencia solidaria	0,71	0,88	0,79	0,80
Prestación de cuidados holísticos	0,71	0,88	0,79	0,81

*Varianza Media Extraída

Los resultados de la validez discriminante del modelo factorial del PCPI-S-VB, según el Criterio de Fornell-Larcker, se presentan en la Tabla 2.

En la Tabla 3 se presentan las Cargas Cruzadas del modelo factorial del *Person-centred Practice Inventory-Staff*.

**Tabla 2-:** Validez Discriminante del modelo factorial del *Person-centred Practice Inventory-Staff*, mediante el Criterio de Fornell-Larcker* (n = 307). Campinas, SP, Brasil, 2023

**Dominio**	**Dominio**																
	**1.**	**2.**	**3.**	**4.**	**5.**	**6.**	**7.**	**8.**	**9.**	**10.**	**11.**	**12.**	**13.**	**14.**	**15.**	**16.**	**17.**
**1.**	**0,73**																
**2.**	0,49	**0,70**															
**3.**	0,41	0,59	**0,74**														
**4.**	0,28	0,41	0,42	**0,79**													
**5.**	0,41	0,49	0,52	0,44	**0,77**												
**6.**	0,30	0,49	0,48	0,36	0,51	**0,77**											
**7.**	0,39	0,30	0,44	0,34	0,49	0,40	**0,79**										
**8.**	0,32	0,29	0,34	0,29	0,43	0,21	0,49	**0,85**									
**9.**	0,38	0,33	0,40	0,30	0,50	0,32	0,57	0,69	**0,78**								
**10.**	0,53	0,43	0,50	0,39	0,49	0,45	0,56	0,49	0,64	**0,71**							
**11.**	0,59	0,49	0,49	0,41	0,46	0,47	0,41	0,39	0,46	0,54	**0,77**						
**12.**	0,32	0,34	0,36	0,32	0,42	0,32	0,56	0,66	0,71	0,56	0,49	**0,79**					
**13.**	0,41	0,47	0,60	0,39	0,50	0,45	0,39	0,41	0,45	0,57	0,60	0,47	**0,75**				
**14.**	0,45	0,39	0,47	0,32	0,44	0,37	0,39	0,33	0,38	0,49	0,54	0,39	0,71	**0,77**			
**15.**	0,38	0,53	0,54	0,41	0,45	0,55	0,35	0,33	0,40	0,50	0,57	0,37	0,67	0,65	**0,79**		
**16.**	0,30	0,49	0,55	0,34	0,41	0,48	0,28	0,35	0,35	0,45	0,51	0,37	0,64	0,61	0,65	**0,84**	
**17.**	0,37	0,51	0,56	0,39	0,42	0,48	0,25	0,28	0,27	0,47	0,51	0,30	0,66	0,56	0,63	0,73	**0,84**

Nota: 1. Competente profesionalmente; 2. Habilidades interpersonales desarrolladas; 3. Comprometido con el trabajo; 4. Autoconocimiento; 5. Claridad de creencias y valores; 6. Combinación de habilidades; 7. Toma de decisiones compartida; 8. Relaciones de equipo eficaces; 9. Distribución de poder; 10. Potencial para la innovación y toma de riesgos; 11. El entorno físico; 12. Sistemas organizacionales de apoyo; 13. Trabajar con las creencias y valores del paciente; 14. Toma de decisiones compartida; 15. Compromiso; 16. Tener presencia solidaria; 17. Prestación de cuidados holísticos

*Valores de la raíz cuadrada de la Varianza Media Extraída en destaque

**Tabla 3-:** Validez Discriminante del modelo factorial del *Person-centred Practice Inventory-Staff*, mediante las Cargas Cruzadas (n = 307). Campinas, SP, Brasil, 2023

Ítems	**1**	**2**	**3**	**4**	**5**	**6**	**7**	**8**	**9**	**10**	**11**	**12**	**13**	**14**	**15**	**16**	**17**
**1**	**0,61**	0,32	0,27	0,17	0,27	0,33	0,21	0,23	0,24	0,27	0,21	0,16	0,27	0,17	0,21	0,17	0,17
**2**	**0,80**	0,41	0,49	0,26	0,25	0,36	0,24	0,20	0,27	0,38	0,29	0,20	0,28	0,23	0,29	0,28	0,33
**3**	**0,77**	0,42	0,51	0,25	0,37	0,30	0,37	0,24	0,29	0,40	0,37	0,23	0,35	0,24	0,34	0,24	0,30
**4**	0,45	**0,65**	0,37	0,22	0,36	0,32	0,13	0,16	0,18	0,22	0,25	0,13	0,24	0,17	0,34	0,30	0,32
**5**	0,39	**0,73**	0,42	0,19	0,32	0,34	0,17	0,11	0,21	0,25	0,28	0,18	0,27	0,23	0,33	0,28	0,34
**6**	0,35	**0,77**	0,47	0,35	0,35	0,37	0,29	0,29	0,29	0,41	0,45	0,30	0,42	0,38	0,44	0,48	0,42
**7**	0,32	**0,65**	0,39	0,38	0,37	0,34	0,21	0,22	0,23	0,29	0,35	0,30	0,36	0,28	0,34	0,28	0,33
**8**	0,45	0,44	**0,67**	0,31	0,29	0,28	0,20	0,18	0,22	0,27	0,28	0,13	0,28	0,20	0,36	0,35	0,33
**9**	0,33	0,41	**0,74**	0,28	0,38	0,27	0,34	0,26	0,30	0,38	0,41	0,32	0,54	0,47	0,42	0,48	0,50
**10**	0,46	0,42	**0,74**	0,32	0,41	0,31	0,35	0,23	0,23	0,41	0,40	0,26	0,46	0,37	0,40	0,39	0,40
**11**	0,47	0,49	**0,79**	0,34	0,44	0,45	0,35	0,30	0,37	0,39	0,32	0,32	0,49	0,34	0,41	0,43	0,44
**12**	0,52	0,44	**0,77**	0,32	0,40	0,46	0,39	0,26	0,34	0,38	0,42	0,28	0,45	0,35	0,41	0,38	0,38
**13**	0,24	0,31	0,35	**0,83**	0,34	0,23	0,34	0,27	0,25	0,32	0,33	0,28	0,34	0,27	0,34	0,26	0,30
**14**	0,21	0,33	0,26	**0,78**	0,32	0,23	0,19	0,22	0,25	0,26	0,33	0,20	0,25	0,26	0,32	0,27	0,25
**15**	0,29	0,34	0,39	**0,76**	0,39	0,39	0,28	0,20	0,22	0,35	0,32	0,28	0,33	0,22	0,32	0,29	0,37
**16**	0,30	0,36	0,43	0,47	**0,75**	0,37	0,38	0,31	0,38	0,36	0,35	0,37	0,43	0,35	0,32	0,36	0,31
**17**	0,25	0,31	0,27	0,25	**0,74**	0,42	0,30	0,26	0,32	0,37	0,35	0,26	0,31	0,29	0,30	0,25	0,30
**18**	0,37	0,45	0,47	0,30	**0,81**	0,39	0,43	0,41	0,45	0,41	0,36	0,33	0,41	0,38	0,41	0,32	0,34
**19**	0,35	0,33	0,34	0,23	0,28	**0,76**	0,23	0,05	0,15	0,26	0,33	0,16	0,32	0,23	0,39	0,30	0,33
**20**	0,35	0,37	0,40	0,29	0,54	**0,83**	0,39	0,25	0,34	0,43	0,41	0,34	0,42	0,33	0,43	0,39	0,39
**21**	0,34	0,44	0,37	0,32	0,33	**0,73**	0,30	0,16	0,23	0,33	0,34	0,23	0,31	0,29	0,46	0,41	0,39
**22**	0,36	0,32	0,40	0,27	0,47	0,45	**0,78**	0,36	0,40	0,43	0,33	0,32	0,33	0,35	0,37	0,24	0,22
**23**	0,26	0,27	0,37	0,35	0,37	0,31	**0,83**	0,38	0,41	0,43	0,35	0,46	0,30	0,29	0,24	0,23	0,21
**24**	0,27	0,20	0,35	0,26	0,38	0,27	**0,80**	0,45	0,50	0,45	0,33	0,55	0,32	0,30	0,25	0,21	0,19
**25**	0,32	0,13	0,27	0,20	0,31	0,23	**0,76**	0,37	0,48	0,47	0,28	0,44	0,29	0,30	0,23	0,19	0,17
**26**	0,33	0,31	0,35	0,28	0,42	0,27	0,45	**0,90**	0,62	0,48	0,33	0,58	0,38	0,29	0,32	0,34	0,28
**27**	0,27	0,27	0,29	0,24	0,36	0,18	0,43	**0,91**	0,63	0,44	0,36	0,60	0,36	0,27	0,29	0,31	0,24
**28**	0,14	0,13	0,20	0,23	0,31	0,06	0,36	**0,74**	0,51	0,31	0,31	0,50	0,32	0,30	0,22	0,25	0,17
**29**	0,25	0,23	0,30	0,16	0,38	0,18	0,35	0,60	**0,78**	0,43	0,34	0,57	0,39	0,29	0,31	0,27	0,19
**30**	0,37	0,27	0,40	0,28	0,46	0,31	0,44	0,41	**0,73**	0,52	0,39	0,42	0,40	0,39	0,44	0,35	0,28
**31**	0,23	0,18	0,22	0,19	0,33	0,23	0,42	0,55	**0,77**	0,47	0,30	0,58	0,23	0,18	0,21	0,18	0,13
**32**	0,29	0,24	0,31	0,29	0,38	0,27	0,54	0,60	**0,83**	0,55	0,39	0,67	0,35	0,29	0,27	0,27	0,22
**33**	0,33	0,25	0,31	0,23	0,36	0,18	0,55	0,57	**0,69**	0,72	0,34	0,62	0,36	0,28	0,30	0,20	0,20
**34**	0,36	0,36	0,33	0,31	0,34	0,44	0,38	0,21	**0,36**	0,72	0,40	0,37	0,43	0,37	0,35	0,38	0,39
**35**	0,34	0,30	0,42	0,30	0,34	0,33	0,26	0,24	**0,30**	0,68	0,41	0,19	0,42	0,41	0,43	0,39	0,42
**36**	0,34	0,34	0,36	0,36	0,23	0,34	0,21	0,23	**0,31**	0,36	0,70	0,24	0,41	0,43	0,44	0,35	0,36
**37**	0,27	0,36	0,37	0,29	0,38	0,38	0,25	0,32	**0,34**	0,40	0,81	0,4	0,47	0,42	0,45	0,41	0,40
**38**	0,33	0,42	0,41	0,31	0,43	0,37	0,46	0,34	**0,40**	0,48	0,80	0,47	0,49	0,40	0,44	0,42	0,42
**39**	0,17	0,19	0,21	0,31	0,28	0,22	0,41	0,50	**0,49**	0,34	0,35	0,71	0,29	0,23	0,23	0,20	0,11
**40**	0,14	0,30	0,23	0,20	0,30	0,18	0,37	0,45	**0,47**	0,33	0,36	0,74	0,29	0,28	0,22	0,23	0,19
**41**	0,25	0,28	0,29	0,30	0,34	0,30	0,46	0,55	**0,57**	0,48	0,4	0,82	0,38	0,34	0,31	0,32	0,25
**42**	0,26	0,3	0,34	0,23	0,37	0,29	0,43	0,54	**0,66**	0,51	0,44	0,83	0,45	0,36	0,36	0,38	0,32
**43**	0,25	0,25	0,31	0,25	0,37	0,27	0,54	0,56	**0,60**	0,51	0,38	0,84	0,44	0,33	0,31	0,29	0,28
**44**	0,35	0,37	0,39	0,29	0,25	0,40	0,24	0,30	**0,33**	0,41	0,38	0,33	0,66	0,45	0,47	0,40	0,42
**45**	0,28	0,34	0,46	0,29	0,33	0,32	0,21	0,26	**0,29**	0,38	0,40	0,29	0,81	0,59	0,56	0,54	0,60
**46**	0,32	0,35	0,51	0,27	0,49	0,31	0,38	0,35	**0,33**	0,49	0,46	0,41	0,76	0,56	0,46	0,54	0,48
**47**	0,30	0,36	0,45	0,32	0,42	0,34	0,33	0,33	**0,39**	0,43	0,55	0,38	0,76	0,52	0,51	0,44	0,48
**48**	0,20	0,31	0,36	0,19	0,36	0,27	0,27	0,27	**0,25**	0,40	0,38	0,26	0,59	0,80	0,53	0,46	0,51
**49**	0,22	0,30	0,42	0,24	0,40	0,30	0,42	0,29	**0,33**	0,39	0,42	0,40	0,55	0,79	0,44	0,45	0,39
**50**	0,26	0,28	0,29	0,31	0,25	0,29	0,19	0,19	**0,29**	0,34	0,44	0,23	0,48	0,71	0,52	0,50	0,40
**51**	0,35	0,46	0,46	0,37	0,31	0,49	0,28	0,24	**0,26**	0,39	0,50	0,25	0,52	0,47	0,79	0,48	0,55
**52**	0,27	0,38	0,35	0,32	0,39	0,33	0,26	0,28	**0,34**	0,42	0,37	0,30	0,49	0,48	0,76	0,45	0,43
**53**	0,30	0,41	0,46	0,29	0,37	0,47	0,28	0,26	**0,35**	0,39	0,48	0,33	0,57	0,58	0,82	0,60	0,52
**54**	0,23	0,42	0,44	0,25	0,34	0,43	0,24	0,34	**0,33**	0,34	0,45	0,34	0,55	0,59	0,64	0,88	0,60
**55**	0,26	0,39	0,47	0,33	0,36	0,39	0,27	0,28	**0,33**	0,40	0,45	0,33	0,61	0,52	0,52	0,86	0,64
**56**	0,33	0,42	0,47	0,29	0,33	0,39	0,18	0,27	**0,22**	0,41	0,39	0,26	0,46	0,42	0,48	0,79	0,60
**57**	0,33	0,37	0,38	0,32	0,29	0,42	0,20	0,16	**0,15**	0,35	0,36	0,14	0,47	0,36	0,44	0,53	0,75
**58**	0,29	0,43	0,48	0,29	0,36	0,38	0,22	0,24	**0,21**	0,41	0,44	0,28	0,58	0,55	0,56	0,64	0,87
**59**	0,34	0,47	0,53	0,37	0,38	0,42	0,21	0,28	**0,30**	0,43	0,48	0,31	0,59	0,49	0,58	0,66	0,90

## Discusión

El proceso de adaptación transcultural del PCPI-S –versión brasileña– siguió las etapas recomendadas internacionalmente^(15-19)^, sin mayores dificultades, debido al compromiso del comité en la evaluación de los 59 ítems, así como a la estructura objetiva y el marco teórico del instrumento^(6-7)^.

Partiendo del supuesto de que la lectura, interpretación y el consecuente llenado de un instrumento no solo generan datos, sino que incitan a los sujetos a reflexionar sobre su práctica y a un posterior movimiento de cambio con el fin de mejorar o ajustar el desempeño, se procuró, durante todo el proceso de traducción y adaptación del instrumento, replicar el constructo original^(15)^.

Con el objetivo de estimular el autoanálisis y la sensibilización de los sujetos, colocándose en la situación presentada por la afirmación, todas las declaraciones fueron readecuadas iniciando con el pronombre “yo”, y se sustituyeron los términos “llevo” y “uso” por “dedico” y “costumbro” en los ítems 13 y 14, respectivamente.

En el análisis del dictamen de los expertos, el término “*challenge*”, presente en los ítems 7 y 17, generó ambigüedades, siendo interpretado por algunos de forma peyorativa, asociándolo a la competencia y a conflictos interpersonales, lo cual es contrario al modelo de cuidado propuesto. Considerando que la afirmación busca investigar la postura del sujeto frente al equipo ante divergencias en las prácticas, se optó, tras consenso entre las investigadoras, el comité de expertos y la lingüista, por sustituir el término por “confrontación”. El término “*ward rounds*”, presente en el ítem 25, fue traducido como “rondas de enfermería”, expresión frecuentemente utilizada en países europeos para describir el relevo de turno al pie de la cama realizado por profesionales de enfermería. Sin embargo, esta terminología no es común en la cultura brasileña, lo que podría generar interpretaciones divergentes entre los participantes. Ante ello, se optó por readecuar el término a “relevo de turno al pie de la cama”, con el fin de facilitar la comprensión de la afirmación.

La validación de contenido del PCPI-S, reconocida internacionalmente como la etapa más relevante en el proceso de validación de instrumentos^(16)^, contó con la amplia experiencia y formación de las participantes del comité de expertos. Se analizaron las evaluaciones de 15 profesionales que actuaban en gestión, docencia e investigación. Como resultado, la versión del PCPI-S obtuvo un IVC superior a 0,87 y un RVC superior a 0,73 para todos los ítems, superando los valores recomendados para el análisis por 15 jueces^(16)^.

Una vez finalizados los ajustes sugeridos por el comité de expertos, se elaboró la versión prefinal del PCPI-S, que posteriormente fue sometida a la prueba piloto. El tiempo medio de respuesta al instrumento fue de 15 minutos, y los participantes consideraron el instrumento adecuado y comprensible.

Tras la finalización de la prueba piloto, se procedió a la etapa de evaluación de las propiedades de medida del instrumento, que comprendió la evaluación de la validez de constructo convergente y discriminante, y la fiabilidad del PCPI-S.

En el análisis factorial confirmatorio, los valores de AVE y de las cargas cruzadas fueron superiores a 0,5, lo que confirmó la validez de constructo convergente, manteniéndose los 17 dominios del PCPI-S original^(17-18)^. Aunque solo el dominio 2 “Habilidades interpersonales desarrolladas” presentó un AVE de 0,49, valor muy próximo al umbral de 0,5 para sugerir validez convergente, todos los ítems del dominio presentaron cargas factoriales superiores a 0,65, lo que también indica evidencia de validez convergente en este dominio^(17-18)^.

El análisis también presentó evidencias de validez discriminante, con base en el Criterio de Fornell-Larcker, en el cual se verifica que los valores de correlación dentro de cada dominio fueron superiores a las correlaciones entre dominios distintos^(17-18)^. En cuanto a la fiabilidad, el análisis de la consistencia interna del instrumento indica que, aunque el alfa de Cronbach presentó valores entre 0,5 y 0,8, este coeficiente es una estimación de la fiabilidad para medir un único constructo común a todos los ítems de un dominio, siempre que sea unidimensional y presente cargas factoriales similares. Como los ítems dentro de un mismo dominio presentaron comportamiento multifactorial, se recurrió a medidas alternativas de fiabilidad, como el omega de McDonald, que varió entre 0,61 y 0,88, y la fiabilidad compuesta, que no asume la unidimensionalidad de los ítems, siendo por tanto una medida más imparcial. La fiabilidad compuesta presentó valores entre 0,77 y 0,89, lo que indica evidencias de consistencia interna del PCPI-S-VB y refuerza la precisión del instrumento^(15,17)^.

A diferencia de la traducción malaya^(10)^, que enfrentó desafíos en la traducción y adaptación debido a la preocupación por mantener la interdisciplinariedad del instrumento, además de haberse realizado en un contexto de atención primaria con una muestra multiprofesional, el proceso de adaptación transcultural al contexto brasileño no presentó dificultades significativas. En la versión malaya, el uso del análisis factorial exploratorio resultó en la exclusión de 6 de los 17 dominios. En cambio, en la adaptación para Brasil fue posible mantener la estructura original del instrumento, en consonancia con las demás traducciones^(7-9,11-14)^.

Conceptualmente, el marco teórico de la práctica centrada en la persona sostiene que la percepción de todas las personas involucradas en el proceso de cuidado debe ser considerada y valorada, incentivando el ejercicio de relaciones armoniosas y, en consecuencia, fortaleciendo el vínculo entre profesionales, familiares y pacientes^(6)^. Así, el uso del PCPI-S-VB puede sensibilizar a los profesionales de la salud sobre dimensiones no técnicas del cuidado, como la experiencia del paciente^(20-23)^. En esta perspectiva, se considera que el uso del instrumento validado en este estudio favorece, entre los profesionales, un ciclo virtuoso de cuidado, integrando las dimensiones afectiva, cognitiva, conductual, moral y empática del cuidado centrado en la persona. La interacción entre estas dimensiones permite que los profesionales compartan y comprendan los sentimientos de la persona enferma, mejorando la comunicación y promoviendo un cuidado más humanizado. De este modo, el deseo de ayudar pasa a ser impulsadopor una motivación interna genuina de los profesionales de la salud^(23)^.

Así, la principal contribución de este estudio es la disponibilidad de la versión brasileña del *Person-centred Practice Inventory-Staff* para sensibilizar a los profesionales de la salud y promover el cuidado centrado en la persona y su familia. La validación del instrumento en otros países^(8-14)^ demuestra su credibilidad, permitiendo la comparabilidad de datos y la identificación de estrategias para el desarrollo de profesionales con competencias clínicas y habilidades conductuales orientadas al cuidado centrado en la persona.

El PCPI-S original fue desarrollado para ser aplicado en diferentes entornos de atención y dirigido al equipo multiprofesional de salud. Por tanto, la principal limitación de este estudio es que la recolección de datos para el análisis de las propiedades de medida de la versión brasileña del PCPI-S se realizó únicamente con profesionales de enfermería en una única institución hospitalaria. Por lo tanto, se recomienda la aplicación de la versión brasileña del PCPI-S en diferentes contextos de atención, involucrando a otros profesionales del equipo multiprofesional.

## Conclusión

La versión brasileña del *Person-centred Practice Inventory-Staff* demostró evidencias de validez y consistencia interna para medir la percepción de la práctica del cuidado centrado en la persona entre profesionales de enfermería en el ámbito hospitalario.

Se reitera la importancia de investigar y validar la percepción de las demás categorías profesionales frente al cuidado centrado en la persona en un escenario muestral más amplio. Considerando que el instrumento puede ser aplicado a diferentes categorías profesionales y en diversos contextos de atención, resulta relevante explorar poblaciones y entornos diversos.

## Data Availability

Todos los datos generados o analizados durante este estudio están incluidos en este artículo publicado.
